# A Novel Bispecific Antibody Targeting PD-L1 and VEGF With Combined Anti-Tumor Activities

**DOI:** 10.3389/fimmu.2021.778978

**Published:** 2021-12-02

**Authors:** Xiaopei Cui, Huifeng Jia, Hong Xin, Lei Zhang, Shi Chen, Simin Xia, Xue Li, Wei Xu, Xiaofang Chen, Yujie Feng, Xiaoyue Wei, Haijia Yu, Yanting Wang, Yifan Zhan, Xiangyang Zhu, Xuemei Zhang

**Affiliations:** ^1^ Department of Pharmacology, School of Pharmacy, Fudan University, Shanghai, China; ^2^ Huabo Biopharma, Member of Zhejiang Huahai Pharmaceutical, Shanghai, China; ^3^ Huaota Biopharma, Member of Zhejiang Huahai Pharmaceutical, Shanghai, China

**Keywords:** bispecific antibodies, VEGF, PD-L1, biological activity, inhibition of cancer growth

## Abstract

Therapeutic monoclonal antibodies (mAbs) blocking immune checkpoints have been mainly used as monotherapy. Recently, combination therapy targeting multiple immune checkpoints has recently been explored to increase anti-cancer efficacy. Particularly, a single molecule targeting more than one checkpoints has been investigated. As dual blocking of PD-1/PD-L1 and VEGF/VEGFR has demonstrated synergism in anti-tumor activities, we developed a novel bispecific antibody, termed HB0025, which is formed *via* fusing the domain 2 of vascular endothelial growth factor receptor 1 (VEGFR1D2) and anti-PD-L1 mAb by using mAb-Trap technology. HB0025 almost completely retains the binding affinities and the biological activities *in-vitro* when compared with the parent anti-PD-L1 mAb or VEGFR1D2 fusion protein. Preclinical data demonstrated that HB0025 was more effective in inhibiting cancer growth than anti PD-L1 mAb or VEGFR1D2 fusion protein. Thus, our bispecific antibody may bring about greater clinical benefits and broader indications.

## Introduction

As the second leading cause of human death globally, malignancies remain a major public health issue (1). According to the data from a study conducted in 2020 with a focus on geographic variability across 20 regions across the world, an estimated 19.3 million new cancer cases and 10.0 million cancer deaths have been estimated (2). Therapeutic antibodies related to blocking the immune escape and angiogenesis have been widely applied in clinical practice with great success (3–5). Particularly, the mAbs targeting programmed cell death protein 1 (PD-1) or programmed cell death-ligand 1 (PD-L1) can reactivate suppressed T-cells to block the cancer-immune escape (6). PD-1 is composed of one immunoglobulin superfamily domain, a 20-amino acid stalk, a transmembrane domain, and a 95-residue intracellular domain (7). It is mainly expressed in activated T-cells, B-cells, natural killer cells, monocytes, and mesenchymal stem cells (8). PD-1 ligand is a 290-amino acid type I transmembrane cell surface glycoprotein encoded by *Cd274* on mouse chromosome 19 and human chromosome 9 (9). PD-L1 is constitutively expressed on DCs, macrophages, mesenchymal stem cells, and bone marrow-derived mast cells (10). PD-L1 has also been found to be expressed by many types of tumor cells (11). The interaction of PD-L1 and PD-1 can inhibit the T-cell response (12). A series of clinical studies have shown that anti-PD-1/PD-L1 antibodies demonstrate robust and long-lasting anti-cancer activities across several solid and hematological cancers, such as lung cancer (13), renal cell cancer (14), melanoma (15), hepatocellular carcinoma (16), and lymphoma (17). Among these, atezolizumab (Tecentriq) is the first immunotherapy drug developed by Roche for PD-L1. The U.S. Food and Drug Administration (FDA) has granted accelerated approval to atezolizumab for use in the treatment of urothelial carcinoma (UC), which was the most common type of bladder cancer recorded in 2016 (18). MAbs or fusion proteins that target vascular endothelial growth factor (VEGF) or VEGF receptor (VEGFR) can also inhibit cancer growth (19). VEGFs can promote mitosis of vascular endothelial cells to form new blood vessels, and they are the most important cytokines in promoting cancer angiogenesis (20–22). The known members of the human VEGF family include VEGF-A, VEGF-B, VEGF-C, VEGF-D, VEGF-E, and placental growth factor (PLGF) (23). VEGF-A has emerged as the single most important regulator of blood vessel formation in health and diseases (24). The VEGFR family is composed of VEGFR1, VEGFR2, and VEGFR3 (21). VEGFR is a class of tyrosine kinase transmembrane glycoproteins; this class consists of an extracellular domain with a meteorite-like early domain, a transmembrane structure, and an intracytoplasmic tyrosine kinase structure (25). Blocking the combination of VEGF and VEGFR1 is expected to effectively inhibit angiogenesis and tumor growth. Bevacizumab (Avastin) was the first antibody approved by the FDA in 2004 for this purpose (26).

However, the occurrence and progression of malignant cancers have been related to several physiological processes, including cell proliferation, induction of angiogenesis, immune escape, resistance to apoptosis, tissue invasion and metastasis, the promotion of inflammatory responses of cancers, and abnormal energy metabolism (27, 28). In many of these cases, the therapeutic efficacy of drugs targeting a single factor is usually reported to be ineffective (29). For example, the response rate of PD-1/PD-L1 mAbs on solid cancers is only 20–30% (30, 31). To overcome these limitations, combination therapy and the use of bispecific antibodies (BsAbs) are of great interest.

BsAbs are man-made antibody-based molecules with two different antigen-binding sites that were first described and identified more than 50 years ago. Research on BsAbs is a rapidly growing field, with a focus on therapy for malignancy and inflammatory diseases (32, 33). The major goal is to simultaneously block two targets involved in the pathophysiological processes toward increasing the therapeutic efficacy (34, 35). Compared to combination therapy with two individual antibodies, BsAbs may potentially increase binding specificity by interacting with two molecules on the same cells; increase local concentration at tumour microenvironment and reduces cost of development and production (36). Currently, several pharmaceutical companies, including Roche, Pfizer, Genentech, Sanofi, AbbVie, and Amgen, are involved in developing BsAb therapeutics (34).

This study reports a novel bispecific antibody, HB0025, which targets both PD-L1 and VEGF and is currently under clinical trial (NCT04678908). The *in vitro* and *in vivo* data from our preclinical studies indicate that this BsAb offers the potential for significant clinical benefits.

## Materials and Methods

### Laboratory Animals, Antibodies, and Cell Lines

Mice used for the present *in vivo* study were 6 - to 8-week-old male NCGs (NOD/ShiLtJGpt-Prkdcem26Cd52Il2rgem26Cd22/Gpt) purchased from GemPharmatech (Nanjing, Jiang Su, China). A375 human melanoma cell line was obtained from the American Type Culture Collection (ATCC, USA) and was maintained *in vitro* as monolayer culture in DMEM medium supplemented with 10% heat inactivated fetal calf serum, 100 U/ml penicillin and 100μg/ml streptomycin; human umbilical vein endothelial cells (HUVEC) were obtained from ATCC; peripheral blood mononuclear cells (PBMCs) used in humanized mice were obtained from SAILYBIO (Shanghai, China); PBMCs used in the mixed lymphoid reaction (MLR) and dendritic cells (DCs) were purchased from Allcells (PCH2019000001, California, USA); CHO-K1-OS8-PD-L1-8D6 cells and Jurkat-NFAT-PD-1-5B8 cells were generated in-house. For HUVEC, culture medium was ECM-1 (CC-4176, Lonza); For CHO-K1-OS8-PD-L1-8D6 cells, culture medium was BalanCD CHO GROWTH A (94120, Irvine Scientific); For PBMCs and DCs used in MLR and Jurkat-NFAT-PD-1-5B8 cells, culture medium was RPMI 1640 (A1049101, Gibco) supplemented with 10% fetal bovine serum (FBS) (10099141C, Gibco). The antibodies, HB0025 (anti-PD-L1/VEGFR1D2-Ig fusion protein), HB002.1T (VEGFR1D2-Ig fusion protein), HB0023 (anti-PD-L1 antibody), and 900543 (anti-Trinitrophenyl antibody, considered as the negative control) were generated also in-house. Bevacizumab was purchased from Roche Diagnostics GmbH and atezolizumab from Genentech.

### Generation, Production, and Structure Characterization of HB0025

The sequence of parent anti-PD-L1 humanized IgG1 antibody (internal code HB0023) was generated by using hybridoma technology. Briefly, human PD-L1-His protein (Sino Biological, #10084-H08H) was emulsified with Freund’s adjuvant, and 5 mice of each strain of BALB/c, CD1, C57BL/6, and SJL were subjected to multi-spot subcutaneous immunization. The serum from the animals was collected on day 35 for PD-L1 titer determination. The spleen lymph node cells from the animals with the highest titers were fused with SP2/0 myeloma cells in a 2:1 ratio by using an electro cell fusion manipulator at day 39 to generate hybridoma cells. Then, the hybridoma cells were screened by ELISA, FACS, and affinity assays. After several rounds of primary screening based on the binding affinity and the blocking activity, one anti-PD-L1 clone was selected. The DNA sequence encoding the variable region of mouse antibody expressed by hybridoma was determined as per the principle of Baobio 5’-RACE technology. Then, the heavy and light chains were constructed by linking the cDNA-cloned mouse VH and VL regions with human IgG1 heavy- and light-constant domains, respectively. The variable region sequence of the antibody was compared with the available sequence in the NCBI protein database. The human framework region deemed suitable for constructing heavy and light chains of CDR transplantation was finally determined through identification and analyses. After the ELISA assay, receptor binding inhibition assay and affinity and cell activity detection, we could obtain a humanized anti-PD-L1 monoclonal antibody showing excellent performance.

To generate the bispecific antibody HB0025, the soluble extracellular domain 2 of human VEGF receptor 1 (VEGFR1D2) was linked to the N-terminus of the heavy chain of HB0023 *via* a (G_2_S)_5_ linker by DNA recombinant technologies, and the sequences of heavy and light chains were sub-cloned into the expression vector, respectively. The sequence of HB002.1T was obtained through a similar method, wherein the VEGFR1D2 was linked to the N-terminus of the IgG-Fc domain.

For the production of HB0023, HB002.1T, and HB0025, the typical monoclonal production methods were employed. Linearized heavy and light plasmids (50 μg in total, HC: LC = 1:2) were co-transfected into CHO cells to derive a stable cell line through the cell line development process. The cell line was then cultured in a shaker for seed expansion until achieving the viable cell density of up to 0.3 × 10^6^ cells/mL, followed by inoculation in the bioreactors for 16-day culturing in a fed-batch mode. The antibodies were captured from the cell supernatant using protein A resin (MabSelect SuRe, Cytiva) and then purified by anion exchange chromatography (Capto Adhere, Cytiva) and cation exchange chromatography (Poros 50HS, Thermo Fisher). The purified HB0025 was characterized for the primary structure, size, and charge variants by liquid chromatography-mass spectrometry (LC-MS), size exclusion chromatography-ultrahigh performance liquid chromatography (SEC-UPLC), and imaged capillary isoelectric focusing (ICIEF).

### Surface Plasma Resonance (SPR) Binding Assays

The SPR studies were performed on the Biacore 8K™ System (Cytiva, Sweden). The sample compartment temperature of the Biacore 8K system and the flow cell temperature were set to 25°C, and the data collection rate was 10 Hz. The anti-human IgG (Fc) antibody (BR-1008-39, Cytiva) was immobilized on a CM5 sensor chip (29149603, Cytiva) to capture the BsAbs as ligand. HB0025 was diluted with running buffer to a concentration that could reach the capture level of 300 RU. The antibodies were flowed over the sensor chip at the rate of 10 μL/min. To measure the binding affinity to VEGF165 protein, the system was firstly injected with 200nM human PD-L1 (449-9AWF1-RA, ACRO Biosystems) to saturate PD-L1 binding by HB0025, then 3-nM human VEGF165 in 2-fold titrations. To measure the binding affinity to PD-L1, the system was firstly injected with 10nM human VEGF165 protein (11066-HNAH, Sino Biological Inc.) to saturate VEGF binding by HB0025, followed by the addition of 50 nM human PD-L1 in 2-fold titrations. Each analyst (PD-L1/VEGF165) was injected for 120 s and dissociated with 1800 s at the rate of 30 μL/min. The sensor chip surfaces were regenerated with 3-M magnesium chloride through a 60-s injection at the rate of 50 μL/min. The binding affinities of the parental antibodies HB0023 and HB002.1T were determined by using the above mentioned method without any enhancement step. The data were analyzed in the single-cycle kinetic system using the capture analysis method, which is pre-defined in the Biacore Insight Evaluation Software. The original data were evaluated in a 1:1 binding model with a fit local kinetics model.

To measure the valence, HB0025, HB0023, and HB002.1T were diluted with the running buffer to a concentration that could reach the capture level (300 RU for HB0025, 250 RU for HB0023, and 200 RU for HB002.1T). Human PD-L1 proteins and human VEGF165 proteins were diluted to a nominal concentration of 200 nM and 10 nM, respectively. The human PD-L1 solution was injected twice at the rate of 10 μL/min for 60 s, and then the human VEGF165 solution was injected twice for 60 s. Finally, the sensor chip surface was regenerated.

The calculations of the binding stoichiometry of HB0025 and PD-L1 and HB0025 and VEGF were fit to the following equations:


$$Conc_{\cdot ligand}=\frac{Response\;Unit(Capture\;level)}{MW_{ligand}}$$

And


$$Conc_{\cdot Analyte}=\frac{Response\;Unit(Analyte\;Level\;in\;total)}{MW_{Analyte}}$$

And


$$Stoichiometric\;Ratio=\frac{Conc_{\cdot Analyte}}{Conc_{\cdot ligand}}$$

Where, MW_ligand_ and MW_Analyte_ are the respective molecular weights (MWs) of the ligand and analyte in the running buffer (pH 7.4). VEGF family proteins are often detected as a disulfide-linked homodimer, and the monomer structure of PD-L1 has been HPLC-verified by the manufacturer ACRO Biosystems. Therefore, the MW of PD-L1 was determined to be 26.0 kDa and that of VEGF 38.4 kDa.

### Enzyme-Linked Immunosorbent Assay (ELISA)

The target-binding affinity of HB0025 was measured by ELISA in the 96-well ELISA microplates (Falcon, Oxnard, CA) coated overnight at room temperature with recombinant human PD-L1 or recombinant human VEGF165 (100 ng/well) in carbonate buffer solution (CBS). The coated plates were blocked with skimmed milk powder diluted to 3% (m/v) in the PBST buffer. HB0025 or bevacizumab/atezolizumab were diluted to 100 μL and then transferred to the plates for incubation at room temperature for 1 h. Then, the plates were washed 5 times with the PBST solution and further incubated with HRP-conjugated goat anti-human Fc antibody at room temperature for 1 h. After incubation, the plates were washed 5 times with the PBST buffer. Tetramethyl benzidine (TMB) solution was then added to the plates for initiating the color reaction. The reaction was stopped with 2 M H_2_SO_4_, and the absorbance was determined using a standard plate reader at 450 nm. The concentration of HB0025 was considered as the abscissa and the absorbance value as the ordinate; the relative binding activity was calculated based on the EC_50_ of the sample.

### Luciferase Reporter Gene System Assays

Jurkat-NFAT-PD-1-5B8 cells are engineered Jurkat cells that stably express human PD-1 and luciferase reporter under the control of an NFAT promoter. CHO-K1-OS8-PD-L1-8D6 cells are engineered CHO-K1 cells that stably co-express PD-L1 and T-cell activator OKT3 scFv. When these two cell types are co-cultured, the PD-1/PD-L1 interaction inhibits TCR signal transduction and NFAT-mediated luciferase activity. The presence of either anti-PD-1 or anti-PD-L1 antibodies can block the PD-1/PD-L1 immunosuppression signal pathway, activate Jurkat cells, NFAT transcription, and induce the luciferase expression.

Jurkat-NFAT-PD-1-5B8 cells and CHO-K1-OS8-PD-L1-8D6 cells were collected and re-suspended in a culture medium at the concentration of 5 × 10^5^/mL and co-seeded in a 96-microwell white plate at the inoculation concentration of 30 μL/well respectively. Serially diluted antibodies were then added into the plates at the concentration of 30 μL/well to achieve the final concentrations of 4000 ng/mL, 1600 ng/mL, 640 ng/mL, 256 ng/mL, 102.40 ng/mL, 40.96 ng/mL, and 16.38 ng/mL. These cells were then incubated at the condition of 5% CO_2_ at 37°C for 6 h. All samples were tested in duplicate. After the incubation, the Bio-GloTM luciferase assay system was applied to estimate the T-cell activation according to the manual (Promega, Madison, WI). Briefly, the plates were equilibrated at room temperature for at least 15 min, followed by the addition of the Bio-GloTM reagent to the plates at the inoculation concentration of 90 μL/well. The reaction plates were incubated at room temperature in the dark for 20 min. The relative luminescence unit (RLU) was detected by using the MD SpectraMax^®^ i3x Microplate Reader scanning at the luminescence unit measure (LUM) endpoint model. Four-parameter fitting was performed with the RLU vs. antibody concentrations using the GraphPad Prism software, and the dose-effect relationship and EC_50_ were determined.

### Mixed Lymphocyte Reaction (MLR) Assays

Human PBMCs and DCs were acquired by using the Easy MLR Kit (Allcells, Alameda). DCs were treated with mitomycin (H19999025, Hisun Pharm Co., Ltd.) for 30 min before co-incubation with human PBMCs. DC cells (50 μL/well, 2.0×10^5^ cells/mL) were co-cultured with PBMCs (100 μL/well, 1.0×10^6^ cells/mL) into a 96-well U-bottom plate. HB0025, atezolizumab, bevacizumab, and 900543 (negative control) were diluted to 100 μL concentration with the RPMI 1640 medium with 10% FBS at the concentration of 3.8 pM-25 nM and added into the co-cultured cells. The plate was incubated at 37°C under a 5% CO_2_ atmosphere for 5 days, followed by centrifugation at 1000 rpm for 10 min to collect the supernatant. The cytokine production (IL-2) in the supernatant was assayed by using the homogeneous time-resolved fluorescence (HTRF) detection method (37).

### Cell Counting Kit-8 (CCK-8) Assays

HUVEC proliferation in response to VEGF165 and the impact of HB0025 on cell proliferation was measured using CCK-8 kits (Dojindo Laboratories, Kumamoto, Japan) as per the manufacturer’s instructions. Briefly, 2000 HUVECs/well were added to a 96-well plate, which was then incubated at 37°C for 2 h, after which 100 μL of the reagent solution containing 20 ng/mL of VEGF165 was added to the cells. Varying amounts of HB0025, bevacizumab or HB002.1T were then added to the plate and the cells were cultured for 72 h at 37°C, followed by the addition of CCK-8 to these plates and incubation for 4 h before spectrophotometric analysis of the absorbance at 450 nm.

### HUVEC Cell Migration Assays

VEGF165 can stimulate HUVEC cell migration. HB0025 can inhibit the process by capturing VEGF165. To measure the cell migration, a 24-well Transwell apparatus was used, and each well contained a 6.5-mm polycarbonate membrane with 8-μm pore size. HUVECs (2 × 10^4^ cells/well) were planted in the top chamber. To the lower chamber, HB0025, 900543 (negative control), atezolizumab, and bevacizumab were added at varying concentrations of 0.01–10 nM. The VEGF165 solution at a constant concentration of 20 ng/mL was added. The transwell microplate was incubated at 37°C under a 5% CO_2_ atmosphere for 24 h. The top chamber was removed, and the cells were fixed with 4% paraformaldehyde (P0099-100, Beyotime) for 20 min, followed by washing with PBS and staining with crystal violet (C0121, Beyotime) for 15 min. After dyeing, the chambers were washed twice with PBS. Five randomly selected fields at the lesion border were acquired using a 10× phase objective on an inverted microscope (Olympus CKX53; Tokyo, Japan). The magnitude of HUVEC migration was evaluated by counting the migrated cells in 5 random fields. One-way analysis of variance (ANOVA; GraphPad Prism) was employed to determine the statistical difference between the groups, and p < 0.05 was considered to indicate a significant difference.

### Pharmacodynamic and Pharmacokinetic Analysis in NCG Mice

Tumor cells (A375 human melanoma cells) were washed twice with PBS and then suspended in a mixture of PBS and Matrigel^®^ (1:1 in volume) to the concentration of 2 × 10^7^/mL. The cells were inoculated subcutaneously in the right flank of each mouse (2 × 10^6^/mouse) for tumor development. Then, 2 × 10^6^ human PBMCs from a healthy donor were injected into each mouse 2 days before A375 tumor inoculation. After the mice were randomized to different treatment groups (n = 10 mice in each group) based on their tumor volume (TVs), with an average TV of 59 mm^3^. The treatment was started 7 days after the tumor inoculation. The treatment groups included vehicle (0.9% saline), HB0025 (3, 6, or 12 mg/kg), HB0023 (5 mg/kg), HB002.1T (2.8 mg/kg), HB0023 combined with HB002.1T (5 mg/kg and 2.8 mg/kg, respectively), bevacizumab (5 mg/kg), atezolizumab (4.8 mg/kg), or bevacizumab combined with atezolizumab (5 mg/kg and 4.8 mg/kg, respectively). All materials were provided through intravenous injections (thrice a week for a total of 8 doses). Tumors were measured in two dimensions using a digital caliper, and the volume is expressed in mm3 using the formula: TV = 0.5 × a × b2, where a and b are the long and short diameters of the tumor, respectively. The tumor volumes were then used to calculate tumor growth inhibition of TV (TGI_TV_) with TGI_TV_ = 1 – (T/C) × %, where T is the average RTV of each treated group, and C is the average RTV of the vehicle control group. RTV is the ratio of tumor volume after administration to pre-dose.

The peripheral blood samples were collected *via* a retro-orbital puncture at different grouping and tumor harvest times. Human CD45^+^ cells were measured by flow cytometry. The tumor tissues of the first 5 mice according to the TVs in vehicle, HB0025, HB0023, HB002.1T, and HB0023+HB002.1T groups were collected and analyzed by multiple immunofluorescence assays. For the assay, formalin fixed and paraffin embedded slides of the tumor tissues were incubated with diluted primary antibody (dilution buffer: SignalStain, 8112, Cell Signaling Technology), overnight at 4°C. HRP-conjugated secondary antibody was added. Then added the fluorophore-conjugated tyramide molecule that served as the HRP substrate. After stripping off the primary/secondary antibody pairs, the antigen-associated fluorescence signal preserved. The processing for antigens were performed sequentially. After the antigen staining cycle, DAPI (4, 6-diamino-2-phenyl indole, 4083, Cell Signaling Technology) solution was added. The information for the primary and secondary antibodies could be found in **Table 1**. The multiplex IHC stained slides were observed under a fluorescent microscope using standard fluorescent filter: CD3 (yellow), CD4 (purple), CD8 (green), CD31 (red), DAPI (blue). 200X images were photographed. The quantitative analysis was processed by HALO soft.

**Table 1:** The information of primary and secondary antibodies used in mIHC detection.

Primary antibodies (Dilution ratio)	Secondary antibodies (Fluorophore-conjugated tyramide molecules)
Rabbit anti-human CD3 antibody (1:100 dilution) (SP162, Abcam, Cambridge, UK)	Anti-rabbit IgG (H+L), F(ab’)2Fragment (Alexa Fluor^®^555 Conjugate) (Cell Signaling Technology)
Rabbit anti-human CD4 antibody (1:100 dilution) (EPR6855, Abcam, Cambridge, UK)	Anti-rabbit IgG (H+L), F(ab’)2Fragment (Alexa Fluor^®^549 Conjugate) (Cell Signaling Technology)
mouse anti-human CD8α antibody (1:100 dilution) (70306s, Cell Signaling Technology)	Anti-mouse IgG (H+L), F(ab’)2Fragment (Alexa Fluor^®^488 Conjugate) (Cell Signaling Technology)
Rabbit anti-human CD31 antibody (1:200 dilution) (92841s, Cell Signaling Technology)	Anti-rabbit IgG (H+L), F(ab’)2Fragment (Alexa Fluor^®^647 Conjugate) (Cell Signaling Technology)

Fluorophore-conjugated tyramide molecules served as the substrate for HRP.

Pharmacokinetics serum samples totaling 120 in numbers were collected from mice receiving vehicle, HB0025 (3, 6, or 12 mg/kg) group at 15 min, 6 h, 12 h, 24 h, 48 h, and 72 h after the last dose inoculation. The WinNonLin non-compartmental model (NCA) was used to calculate the pharmacokinetic parameters of HB0025. The calculations of area under the curve (AUC_last_), area under the moment curve (AUMC_last_) and mean residence time (MRT_last_) were fit to the equations,


$$AUC_{last}=\int_{0}^{last}C_{p}dt$$

And


$$AUMC_{last}=\int_{0}^{last}tC_{p}dt$$

And


$$MRT_{last}=\frac{AUMC_{last}}{AUC_{last}}$$

Where, C_p_ is the analyte concentration in the plasma and t was the experimental time.

### Statistical Analyses

The results are presented as mean ± standard error of the mean (SEM). p < 0.05 was considered to indicate statistical significance (* p < 0.05, ** p < 0.01, and *** p < 0.001). The differences in the TVs between the compared groups were analyzed by the IBM SPSS Statistics 25.0 software using the one-way ANOVA, followed by the least significant difference multiple comparison test.

## Results

### Generation and Characterization of Bispecific Antibody Dual Targeting PD-L1 and VEGF

**Figure 1:**
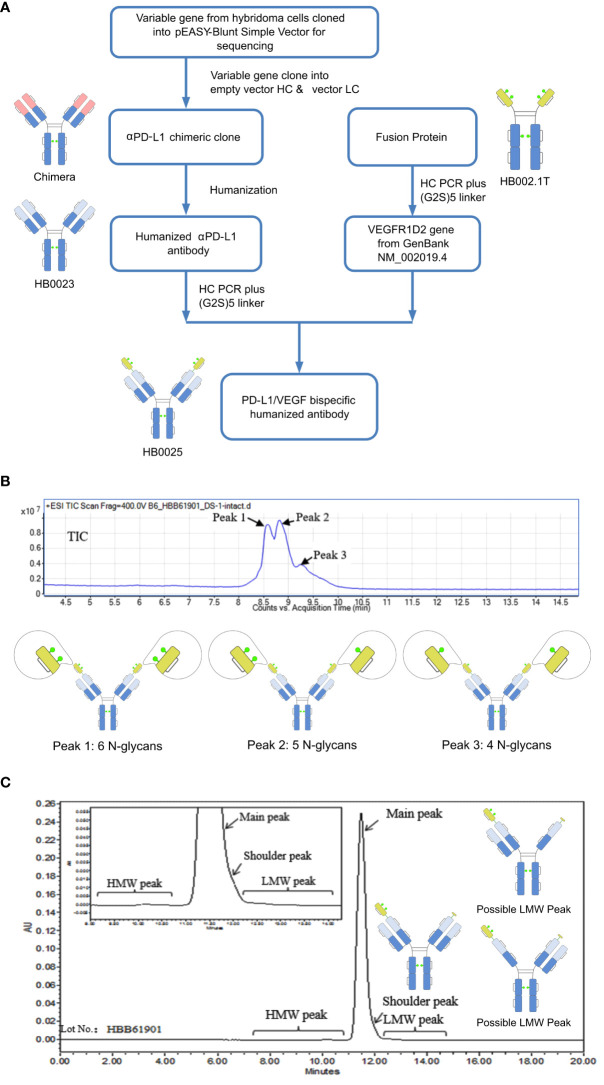
A depiction of the generation process and structure characterization of HB0025. **(A)** Schematic representation of the generation process for the bispecific antibody and the parent molecules. **(B)** Mass analysis of HB0025 and the related schematic presentation of the peaks. **(C)** SEC-UPLC analysis of HB0025 and the related schematic presentation for the shoulder peak and low molecular weight peaks.

The protein HB0025 is a bispecific antibody that targets both PD-L1 and VEGF. The parent anti-PD-L1 humanized IgG1 antibody (internal code HB0023) was generated by using the hybridoma technology (38). HB0023 has a high binding affinity and specificity to PD-L1 and PD-1/PD-L1 blocking activity. To generate the bispecific antibody HB0025, the soluble extracellular domain 2 of VEGFR1D2 was fused to the N-terminus of the heavy chain of HB0023 *via* a (G_2_S)_5_ linker. The (G_2_S)_5_ linker consists of 5 repeats of the amino acid sequence “GGS” (**Figure 1A**).

The molecular formula of HB0025 is C_7530_H_11690_N_2026_O_2338_S_56_, with a theoretical MW of approximately 170.8 kDa (De-glycan Mass). To characterize the primary structure, reversed-phase chromatography-mass spectrometry (RP-MS) was performed. The intact MW was obtained by deconvolution of the mass spectrum. Three peaks (peak 1, peak 2, and peak 3) were observed in the total ion chromatogram (TIC) map and confirmed in de-convoluted mass spectra (**Figure 1B** and **Supplementary Data 1**). The average MWs of peaks 1, 2, and 3 were approximately 182, 180, and 178 kDa, respectively (**Table 2**). After de-glycosylating through incubation with PNGase F, only one main peak with a MW of 170 kDa was detected. Three N-glycosylation sites existed on each heavy chain: N36, N68 (on the VEGFR1D2 domain), and N415 (on the FC region of the heavy chain, equivalent to the N297 conserved site of monoclonal antibody). Among the three sites, the modification of N68 was partial, while that of the remaining two sites N36 and N415 were complete. Therefore, two types of heavy chains were detected: one with 3 glycan groups and the other with 2 glycan groups. After the heavy chains were randomly paired, three types of proteins containing 6, 5, and 4 glycan groups, respectively, were formed (**Figure 1B**).

**Table 2:** Theoretical and measured mass of intact HB0025.

Peak	Glycosylation	Theoretical Mass (kDa)	Measured Mass (kDa)	Mass error (kDa)
**Peak 1**	G2FS1(4)/G0F/G1F	182131.1	182134.2	3.1
	G2FS1(4)/G1F(2)	182293.2	182289.5	-3.7
	G2FS1(3)/G2FS2/G0F/G1F	182422.4	182426.2	3.8
	G2FS1(3)/G2FS2/G1F(2)	182584.5	182585.2	0.7
**Peak 2**	G2FS1(3)/G0F/G1F	180070.3	180073.6	3.3
	G2FS1(3)/G1F(2)	180232.4	180217.2	-15.2
	G2FS1(2)/G2FS2/G0F/G1F	180361.5	180366.4	4.9
	G2FS1(2)/G2FS2/G1F(2)	180523.6	180512.8	-10.8
**Peak 3**	G2FS1(2)/G0F/G1F	178009.4	178014.0	4.6
	G2FS1(2)/G1F(2)	178171.6	178169.8	-1.8
	G2FS1/G2FS2/G0F/G1F	178300.6	178306.7	6.1
	G2FS1/G2FS2/G1F(2)	178462.8	178467.1	4.3

(2), (3), and (4) indicate that there are 2, 3, and 4 N-glycan.

The SEC-UPLC was employed to determine the size-based purity. As shown in **Figure 1C**, the main peak ratio reached 97.0%, the high molecular weight (HWM) peak ratio was 0.3%, the shoulder peak ratio was about 2.0% and the low molecular weight (LWM) peak ratio was 0.7%. Size exclusion chromatography-multi-angle static light scattering (SEC-MALS) combined analysis was performed to analyze the MW of the main component and the high MW component. The molecule weight of the HMW peak was approximately 642 KDa, which was approximately 3.5-times that of the main peak. This result supported the speculation that the high MW component could be a trimer (**Supplementary Data 1**). The shoulder peaks in the SEC-UPLC spectrum were collected and measured by RP-MS. The measured MW of the shoulder peak was consistent with the theoretical HC MW, that is, one VEGFR domain resulting from the cleavage between N-terminal asparagine 99 and threonine 100 of the VEGFR domain was missing (**Figure 1C**). Moreover, the proportion was too low to collect for the low MW analysis; the hypothesized molecular structure is depicted in **Figure 1C**. The charge variant distribution was identified by iCIEF, and the acid and basic peaks showed no affinity difference when compared with the main peaks (**Supplementary Data 1**).

### HB0025 Demonstrates Independent Binding Activity of the Two Targets

To confirm that the targets did not interfere with each other, we employed the SPR method to quantify the antigen-antibody binding ratio. When compared with the stoichiometric ratio of samples binding to PD-L1 and VEGF, no difference was detected in the contact order of the samples exposed to antigens. Furthermore, one HB0025 was found to bind to approximately 1.7 PD-L1 monomers and 1.2 VEGF dimers simultaneously, showing similar binding properties to its parent molecules HB0023 and HB002.1T (**Table 3** and **Supplementary Table 1**). In this study, the interaction model demonstrated its independence when binding to both the targets simultaneously. This result also provides theoretical support to the combined effect.

**Table 3:** Kinetic results of samples binding to PD-L1 and VEGF with a 1:1 binding model.

Ligand	Enhancement	Analyte	Kinetics Chi^2^ (RU^2^)	Ka (1/Ms)	Kd (1/s)	KD (M)
HB0025	Buffer	VEGF165	4.30E-01	1.44E+08	6.81E-04	4.72E-12
	PD-L1	VEGF165	2.31E-01	1.48E+08	4.41E-04	2.98E-12
	Buffer	PD-L1	1.18E-01	2.90E+05	5.10E-04	1.76E-09
	VEGF165	PD-L1	6.02E-02	2.00E+05	3.95E-04	1.98E-09
HB002.1T	\	VEGF165	2.01E-01	4.54E+07	5.03E-05	1.11E-12
HB0023	\	PD-L1	9.77E-02	2.34E+05	5.25E-04	2.24E-09
Bevacizumab	\	VEGF165	1.91E-02	1.41E+06	6.17E-04	4.39E-10
Atezolizumab	\	PD-L1	9.22E-02	1.04E+06	5.01E-04	4.82E-10

### HB0025 Is a Potent Dual Inhibitor of VEGF and PD-L1-Mediated Activity *In Vitro*

**Figure 2:**
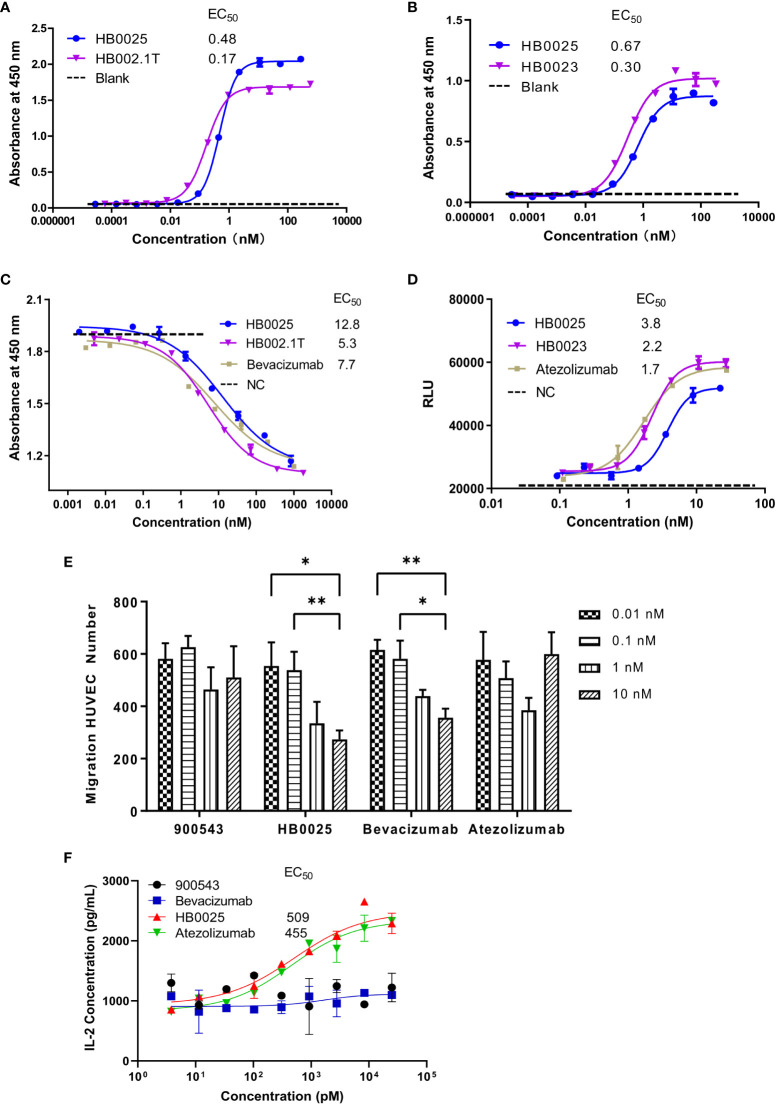
The profile of binding affinity and blocking activity of HB0025, the parent molecules, and the market antibodies. **(A)** The ELISA binding activity of HB0025 and HB002.1T to VEGF. **(B)** The ELISA binding activity of HB0025 and HB0023 to PD-L1. The PBST was used as a blank sample. **(C)** The blockade effect of HB0025, HB002.1T, and bevacizumab on VEGF-induced HUVEC proliferation by the CCK-8 assay. Negative control (NC): The cells were treated with the mixture of VEGF solution and detection medium in the ratio of 1:1. **(D)** Inhibitory effect of HB0025, HB0023, and atezolizumab on the PD-L1 pathway by the luciferase reporter gene system. Negative control (NC): The cells were treated with a second antibody alone. **(E)** Inhibition of HUVEC migration by 900543 (negative control), HB0025, atezolizumab and bevacizumab (positive control); 0.01 nM and 0.1 nM were compared with 10 nM, *p < 0.05, **p < 0.01. **(F)** The dose-effect fitting curve of antibodies on the recovery of human IL-2 secretion, taking 900543 as the negative control. The data are representative of mean ± SEM; Experiments of **(A–D, F)** were performed in duplicate; data of Experiment E were derived from 5 visual fields.

The binding affinities of HB0025 to VEGF and PD-L1 were determined and compared to those of the parent anti-PD-L1 mAb (internal code HB0023) and VEGFR1 domain-Fc fusion protein (internal code HB002.1T), respectively. As for VEGF, the EC_50_ was determined by ELISA for HB0025 and HB002.1T to be 0.48 and 0.17 nM, respectively. As to PD-L1, the EC_50_ for HB0025 and HB0023 were 0.67 nM and 0.30 nM, respectively. HB0025 showed a slight increase in EC_50_ when compared with that of the parent anti-PD-L1 antibody and VEGFR1D2 domain-Fc fusion protein (**Figures 2A, B**). The SPR analysis data revealed that HB0025 had comparable affinities with the parent molecules (**Table 3**).

HUVEC that over-expresses VEGFR on the membrane surface and is sensitive to VEGF-promoted cell proliferation was used to investigate the inhibitory properties of HB0025 on VEGF-mediated effect on proliferation. The CCK-8 assay results demonstrated that HB0025, HB002.1T, and bevacizumab had comparable potencies toward inhibiting the HUVEC proliferation (**Figure 2C**). A luciferase reporter gene system for NAFT activation was employed for the investigation of the inhibitory effect of HB0025 on the PD-L1 pathway *in vitro*. Co-culture of CHO-K1-OS8-PD-L1-8D6 cells (target cells overexpressing PD-L1) and Jurkat-NFat-PD-1-5B8 cells (activated effector T-cells) were employed to detect the inhibitory effect of HB0025 on the PD-L1 pathway. These results together demonstrated that HB0025, HB0023, and atezolizumab had comparable potencies in inhibiting the biological activity of PD-L1 (**Figure 2D**).

To evaluate the blocking capability of HB0025 on VEGF-mediated cell migration, the migration inhibition rate of HUVECs was tested. The migration ratio showed obvious inhibition when HB0025 and bevacizumab were added to the lower chamber. A dose-dependent effect of HB0025 and bevacizumab was observed. In this study, HB0025 prevented cell migration and demonstrated an inhibitory potency comparable to that of bevacizumab (**Figure 2E**). MLR was performed to evaluate the blocking efficiency of HB0025 on PD-1/PD-L1 interaction. Both HB0025 and atezolizumab increased the secretion of IL-2 in a dose-dependent manner in the culture of allogenic monocyte-derived dendritic cells and T-cell containing PBMCs (**Figure 2F**). The EC_50_ of the functional activity of HB0025 was approximately 509 pM, while that of atezolizumab was approximately 455 pM.

Altogether, our data suggest that the HB0025 largely maintains the binding and blocking activities of both the parent molecules and is a potent dual inhibitor for both VEGF- and PD-L1-mediated activities *in vitro*.

### HB0025 Exhibits Combined Anti-Tumor Activities *In Vivo*

**Figure 3:**
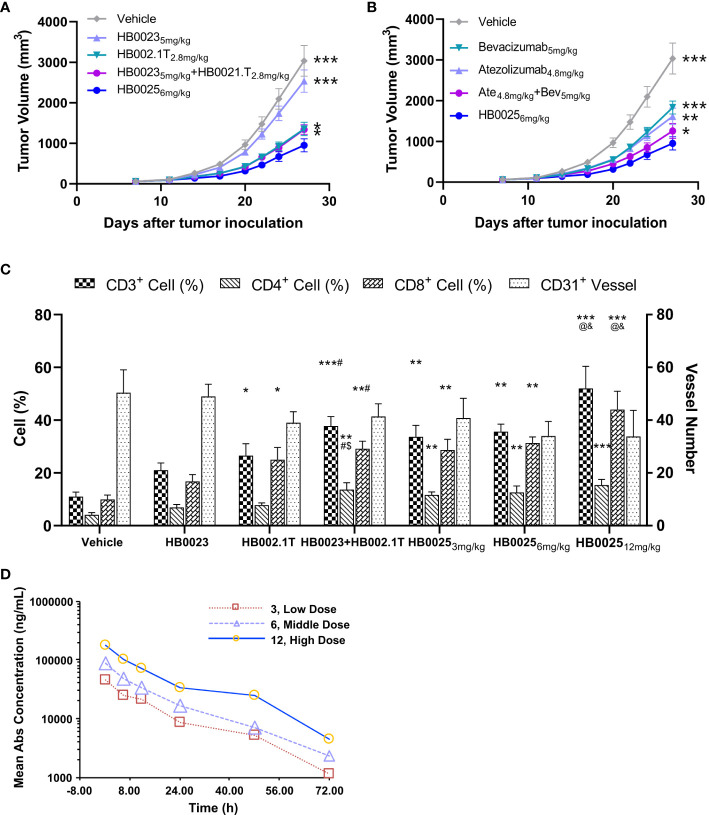
Pharmacodynamics and pharmacokinetic analysis in NCG mice. **(A)** HB0025 treatment compared with HB0023, HB002.1T, and HB0023+HB002.1T in NCG mice. **(B)** HB0025 treatment compared with bevacizumab, atezolizumab, and bevacizumab+atezolizumab in NCG mice. *p < 0.05, **p < 0.01, ***p < 0.001 compared with HB0025_6mg/kg._ **(C)** Tumor-infiltrating lymphocytes (CD3^+^, CD4^+^, CD8^+^) and the numbers of blood vessels (CD31^+^). *p < 0.05, **p < 0.01, ***p < 0.001 compared with the vehicle; ^#^p < 0.05 compared HB0023 with HB0023+HB002.1T; ^$^p < 0.05 compared HB002.1T with HB0023+HB002.1T; ^@^p < 0.05 compared with HB0025 (3 mg/kg)/HB0025 (6 mg/kg) with HB0025 (12 mg/kg); and p < 0.05 compared HB002.1T+HB0023 with HB0025 (12 mg/kg). **(D)** The pharmacokinetic profiles of HB0025 in NCG mice at doses 3, 6, and 12 mg/kg. data expressed as mean ± SEM.

The anti-cancer activities of HB0025 were evaluated in a subcutaneous A375 human melanoma xenograft model using PBMC humanized NCG mice. The peripheral blood of humanized mice was collected to analyze the reconstitution of human CD45^+^ cells. At the time of grouping, the proportion of human CD45^+^ cells was >1%, while that at the end of the experiment was >60% (**Supplementary Table 2**). The xenograft cancer model was treated with HB0025 as well as the control drugs HB0023, HB002.1T, atezolizumab, and bevacizumab at equal mole dose through intravenous injections. The TVs were measured thrice a week. The calculated tumor growth inhibition of TV (TGI_TV_) was higher in the HB0025-treatment groups with 3, 6, or 12 mg/kg (66%, 69%, and 67%, respectively) than in those treated with HB0023_5mg/kg_ (17%), HB002.1T_2.8mg/kg_ (54%), HB0023_5mg/kg_ + HB002.1T_2.8mg/kg_ (55%), bevacizumab_5mg/kg_ (38%), atezolizumab_4.8mg/kg_ (46%), and atezolizumab_4.8mg/kg_ + bevacizumab_5mg/kg_ (59%). Notably, the anti-tumor efficacy of HB0025 was stronger at the equivalent molar dose than that of the combination of single functional antibodies HB0023 and HB002.1T (p < 0.05) (**Figure 3A**, **Supplementary Figure 1** and **Supplementary Table 3**) and the combination treatment of anti-PD-L1 antibody atezolizumab and anti-VEGF antibody bevacizumab on the market (p < 0.05) (**Figure 3B**). As shown in **Figure 3C**, HB0025 treatment dose-dependently increased the number of infiltrating CD3^+^ and CD8^+^ T-cells within tumors when compared to that in vehicle-treated cells. The number of CD31^+^ blood vessels found within tumours were lower than vehicle in all VEGF-targeted groups, though not significantly different (**Supplementary Figure 2** and **Supplementary Table 4**). These data demonstrate that treatment with HB0025 induced activation and recruitment of cytotoxic T-cells to inhibit tumor growth.

### HB0025 Has a Dose-Dependent Pharmacokinetic Profile

The pharmacokinetic properties of HB0025 at different doses (3, 6, and 12 mg/kg) were examined in male NCG mice (and same with *in-vivo* efficacy model, n = 5). The plasma was collected at different time points after intravenous injection and analyzed by ELISA. The drug helped achieve homeostasis in the body after 8 rounds of three doses in a week. The dose ratio for the low-, medium-, and the high-dose group was 1:2:4, while the ratio of average the peak drug concentration in serum (C_max_), and the drug exposure area under the curve (AUC_last_) were 1:1.90:3.89 and 1:1.75:3.99, respectively. The mean residence time (MRT_last_) was not significantly different among the different dose treatment groups. In the dose range of 3–12 mg/kg, HB0025 was linearly metabolized in cancer-bearing mice (**Figure 3D**). These results indicate that the systemic exposure of HB0025 was proportional to the administered dosage.

## Discussion

In the past decade, targeting more than one immune checkpoint with a single molecule has become one of the most attractive drug development areas that have emerged as an alternative to combination therapy or the use of mixture (36). The promise of bi-functional molecule therapeutics has prompted the design of various formats of antibody-like proteins that can be tailored to specific needs (39, 40). This study reports a novel IgG-like bi-functional molecule termed HB0025. HB0025 could concurrently block both the PD-L1 and VEGF pathways and demonstrated combined anti-cancer activities both *in vitro* and *in vivo* when compared with each of the parent drug candidates.

To develop bi-functional molecules, the key properties of the parent molecules must be maintained. At the molecular level, the SPR affinity assay data demonstrated that HB0025 adequately retained the affinities to both targets and that there was no mutual interference in ligand binding between the two targets. Functionally, HB0025, as the PD-L1 blocker, was as effective as the parental anti-PD-L1 or the therapeutic antibody, atezolizumab. HB0025 also retained similar functional activities to block VEGF-mediated cell proliferation and migration when compared to that of the anti-VEGF parent protein and bevacizumab.

**Figure 4:**
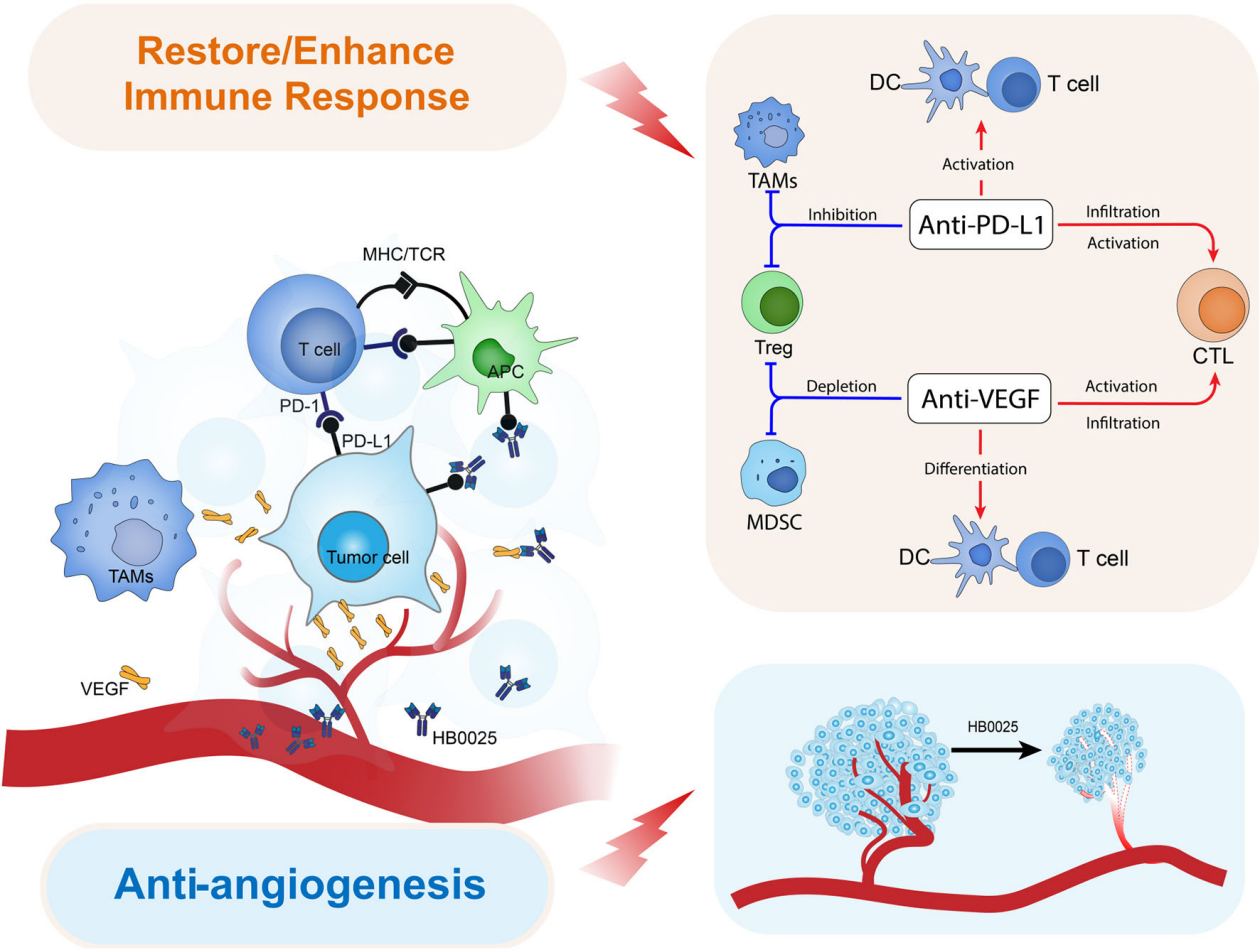
Model diagram of HB0025 biological mechanism *in vivo*.

Preclinical studies have demonstrated that, after multiple administrations of VEGF inhibitors, the tumor inhibition was slowed down because of the PD-L1 high expression (41). Combination therapy targeting both PD-1/PD-L1 and VEGF has been suggested to have better anti-tumor effects compared to that with single drugs (42). Currently, multiple combination therapies targeting PD-1/PD-L1 (mAbs) and VEGF (mAbs or small molecule tyrosine kinase inhibitor) have been approved for clinical trials for diverse indications. Clinical trial results so far have demonstrated the advantages of PD-L1+VEGF dual targets therapy that can significantly reduce the risk of death and disease progression as well as improve the survival rate (43, 44). In humanized mice grafted with human tumor cells, HB0025 showed a higher tumor suppression activity relative to that of the parent molecules and that of the combination therapy of the two-parent drug candidates. The potential mechanisms for the combined anti-tumor activity of HB0025 are illustrated as follows (**Figure 4**): 1) HB0025 could block the interaction between PD-1 and PD-L1 and enhance the T-cell responses (45). 2) HB0025 could trap VEGF-A and VEGF-B and reduce its binding to VEGFR, which further blocked the PI3K/AKT/mTOR signaling pathway and decreased the proliferation of HUVEC (46). 3) HB0025 could also improve the T-cell function by inhibiting the function of VEGF and promoting the infiltration of T-cells into cancer, thereby reversing the immunosuppressive cancer microenvironment into an immune-activated state (44, 47). 4) When compared to combination therapy, coupling anti-VEGF and PD-L1 could increase the local drug concentration in a tumor microenvironment by targeting PD-L1 expressed by tumor cells.

Achieving effective concentrations within solid cancer masses has been challenging for large molecule drugs (48). Better penetration and suitable retention in the targeted area of the body are therefore ideal parameters for large molecule drugs to reach an optimal therapeutic efficacy. For a given protein drug, the rate of diffusion through cancers is inversely correlated to its MW, charge, and valence (49, 50). HB0025 has a MW of approximately 180.2 kDa, which is slightly greater than that of a mAb, and has a pI of 8.40–9.26, which makes it beneficial for penetration. In the dose range of 3–12 mg/kg, HB0025 was linearly metabolized in cancer-bearing mice. Taken together, our results signify the good pharmacokinetic profile of HB0025.

In conclusion, the stable bispecific antibody HB0025, which targets both PD-L1 and VEGF, possesses suitable pharmacodynamics and pharmacokinetic profiles to become a clinical drug candidate. HB0025 offers IgG-like biophysical properties that are acceptable for pharmaceutical development and that allow concurrent blockage of two pathways to enhance its anti-cancer activities. Collectively, our data support further clinical studies with HB0025.

## Data Availability Statement

The original contributions presented in the study are included in the article/**Supplementary Material**. Further inquiries can be directed to the corresponding authors.

## Ethics Statement

The animal study was reviewed and approved by Ethics Committee of Yikang (Beijing) Medical Technology Co., Ltd.

## Author Contributions

Authors XCu, HJ, HX, HY, XW, YZ, XZ and XZ made substantial contributions to molecular design and study conception; Authors SC, XL and YW made contributions to in-vitro validation; LZ and WX made contributions to animal studies and interpretation of the data; SX, XCh and YF made contributions to the product production and analysis. All authors contributed to the article and approved the submitted version.

## Funding

This work was supported by grants from the Shanghai Science and Technology Innovation (NO. 20S11905000 to XZhang) and the Clinical Research Plan of SHCD.

## Conflict of Interest

Authors XCu, HJ, SC, SX, XL, WX, XCh, YF, XW, HY, YW, YZ XZ were employed by company Huabo Biopharma and authors LZ and XZ were employed by Huaota Biopharma.

The remaining author declares that the research was conducted in the absence of any commercial or financial relationships that could be construed as a potential conflict of interest.

## Publisher’s Note

All claims expressed in this article are solely those of the authors and do not necessarily represent those of their affiliated organizations, or those of the publisher, the editors and the reviewers. Any product that may be evaluated in this article, or claim that may be made by its manufacturer, is not guaranteed or endorsed by the publisher.

## References

[1] Yang R Zhou Y Wang Y Du C Wu Y. Trends in Cancer Incidence and Mortality Rates in the United States From 1975 to 2016. Ann Transl Med (2020) 8(24):1671–1. doi: 10.21037/atm-20-7841 PMC7812176 33490183

[2] Hyuna S Jacques F Rebecca LS Mathieu L Isabelle S Ahmedin J. Global Cancer Statistics 2020: GLOBOCAN Estimates of Incidence and Mortality Worldwide for 36 Cancers in 185 Countries. CA-Cancer J Clin (2020) 3(71):209–49. doi: 10.3322/caac.21660 33538338

[3] Anthony L Shawn A Alan S Tien H. Recent Progress in Therapeutic Antibodies for Cancer Immunotherapy. Curr Opin Chem Biol (2018) 44:56–65. doi: 10.1016/j.cbpa.2018.05.006 29885949

[4] Liu K Ren T Huang Y Sun K Bao X Wang S. Apatinib Promotes Autophagy and Apoptosis Through VEGFR2/STAT3/BCL-2 Signaling in Osteosarcoma. Cell Death Dis (2017) 8(8):e3015. doi: 10.1038/cddis.2017.422 28837148 PMC5596600

[5] Cheng Y Wang Q Li K Shi J Wu L Han B. OA13.03 Anlotinib as Third-Line or Further-Line Treatment in Relapsed SCLC: A Multicenter, Randomized, Double-Blind Phase 2 Trial. J Thorac Oncol (2018) 13(10):S351–2. doi: 10.1016/j.jtho.2018.08.308

[6] Arghya R Deepika SD Yan S Paul R Nikhil CM Dharminder C. Targeting PD1–PDL1 Immune Checkpoint in Plasmacytoid Dendritic Cell Interactions With T Cells, Natural Killer Cells and Multiple Myeloma Cells. Leukemia (2015) 29:1441–4. doi: 10.1038/leu.2015.11 PMC5703039 25634684

[7] Freeman GJ Sharpe AH Keir ME Butte MJ. PD-1 and Its Ligands in Tolerance and Immunity. Annu Rev Immunol (2008) 26(1):677–704. doi: 10.1146/annurev.immunol.26.021607.090331 18173375 PMC10637733

[8] Sharpe AH Freeman GJ. The B7-CD28 Superfamily. Nat Rev Immunol (2002) 2(2):116–26. doi: 10.1038/nri727 11910893

[9] Budczies J Denkert C GyőRffy B Schirmacher P Stenzinger A. Chromosome 9p Copy Number Gains Involving PD-L1 Are Associated With a Specific Proliferation and Immune-Modulating Gene Expression Program Active Across Major Cancer Types. BMC Med Genomics (2017) 10(1):74. doi: 10.1186/s12920-017-0308-8 29212506 PMC5719741

[10] Yamazaki T Akiba H Iwai H Matsuda H Aoki M Tanno Y. Expression of Programmed Death 1 Ligands by Murine T Cells and APC. J Immunol (2002) 169(10):5538–45. doi: 10.4049/jimmunol.169.10.5538 12421930

[11] Drew MP. The Blockade of Immune Checkpoints in Cancer Immunotherapy. Nat Rev Cancer (2012) 12:252–64. doi: 10.1038/nrc3239 PMC4856023 22437870

[12] Amarnath S Mangus CW Wang J Fang W Fowler DH. The PDL1-PD1 Axis Converts Human TH1 Cells Into Regulatory T Cells. Sci Transl Med (2011) 3(111):111ra120. doi: 10.1126/scitranslmed.3003130 PMC3235958 22133721

[13] Naiyer AR Matthew DH Alexandra S Pia K Vladimir M Jonathan JH. Cancer Immunology. Mutational Landscape Determines Sensitivity to PD-1 Blockade in Non-Small Cell Lung Cancer. Science (2015) 348(6230):124–8. doi: 10.1126/science.aaa1348 PMC4993154 25765070

[14] Kathryn EB Johnson DB Sosman JA. PD-1/PD-L1 Blockade in Renal Cell Cancer. Expert Rev Clin Immunol (2017) 13(1):77–84. doi: 10.1080/1744666X.2016.1214575 27426025 PMC5555220

[15] Katy KT Adi ID. The Role of Anti-PD-1/PD-L1 Agents in Melanoma: Progress to Date. Drugs (2015) 75:563–75. doi: 10.1007/s40265-015-0376-z 25802230

[16] Xue H Jinhua H. The Research Progress of PD-1/PD-L1 in the Treatment of Hepatocellular Carcinoma. J Cancer Control Treat (2017) 44(14):726–30. doi: 10.3969/j.issn.1674-0904

[17] Yanxia H Jun Y Jiaqi W Yanru W Qingyuan Z Yue Z. Research Progress of PD-1/PD-L1 Inhibitor in the Treatment of Lymphoma. J Modern Oncol (2018) 26(20):3318–21. doi: 10.3969/j.issn.1672-4992.2018.20.036

[18] Ning YM Suzman D Maher VE Zhang L Tang S Ricks T. FDA Approval Summary: Atezolizumab for the Treatment of Patients With Progressive Advanced Urothelial Carcinoma After Platinum-Containing Chemotherapy. Oncologist (2017) 22(6):743–9. doi: 10.1634/theoncologist.2017-0087 PMC5469588 28424325

[19] Atzori MG Tentori L Ruffini F Ceci C Bonanno E Scimeca M. The Anti-Vascular Endothelial Growth Factor Receptor-1 Monoclonal Antibody D16f7 Inhibits Glioma Growth and Angiogenesis *In Vivo* . J Pharmacol Exp Ther (2017) 364(1):77–86. doi: 10.1124/jpet.117.244434 29025978

[20] Oku T Tjuvajev JG Miyagawa T Sasajima T Joshi A Joshi R. Tumor Growth Modulation by Sense and Antisense Vascular Endothelial Growth Factor Gene Expression: Effects on Angiogenesis, Vascular Permeability, Blood Volume, Blood Flow, Fluorodeoxyglucose Uptake, and Proliferation of Human Melanoma Intracerebral Xenogr. Cancer Res (1998) 58(18):4185–92. 9751633

[21] Ferrara N Gerber HP Lecouter J. The Biology of VEGF and Its Receptors. Nat Med (2003) 9(6):669–76. doi: 10.1038/nm0603-669 12778165

[22] Vitt UA Hsu SY Hsueh A. Evolution and Classification of Cystine Knot-Containing Hormones and Related Extracellular Signaling Molecules. Mol Endocrinol (2001) 15(5):681–94. doi: 10.1210/mend.15.5.0639 11328851

[23] Holmes DI Zachary I. The Vascular Endothelial Growth Factor (VEGF) Family: Angiogenic Factors in Health and Disease. Genome Biol (2005) 6(2):209. doi: 10.1186/gb-2005-6-2-209 15693956 PMC551528

[24] Ferrara N Mass RD Campa C Kim R. Targeting VEGF-A to Treat Cancer and Age-Related Macular Degeneration. Annu Rev Med (2007) 58(1):491–504. doi: 10.1146/annurev.med.58.061705.145635 17052163

[25] Alberto Á-A Muhl L Gaengel K. VEGF Receptor Tyrosine Kinases: Key Regulators of Vascular Function. Curr Top Dev Biol (2016) 123:433–82. doi: 10.1016/bs.ctdb.2016.10.001 28236974

[26] Mukherji SK. Bevacizumab (Avastin). Am J Neuroradiology (2010) 31:235–6. doi: 10.3174/ajnr.A1987 PMC7964163 20037132

[27] Falcon BL Pietras K Chou J Chen D Sennino B Hanahan D. Increased Vascular Delivery and Efficacy of Chemotherapy After Inhibition of Platelet-Derived Growth Factor-B. Am J Pathol (2011) 178(6):2920–30. doi: 10.1016/j.ajpath.2011.02.019 PMC3124291 21641409

[28] Sabina S Paola M Agnoli C Franco B Valeria P Sara G. Prospective Study on the Role of Glucose Metabolism in Breast Cancer Occurrence. Int J Cancers (2011) 4:921–9. doi: 10.1002/ijc.26071 21413010

[29] Generoso U. Lesson From Acute Experimental Pancreatitis: Multidrug Strategies Is Effective Than Single-Target Therapy. J Pancreas (2012) 13:543–4. doi: 10.6092/1590-8577/1210 22964964

[30] Youhai J Zhao X Jing F Hongyang W. Progress and Challenges in Precise Treatment of Tumors With PD-1/PD-L1 Blockade. Front In Immunol (2020) 11:339. doi: 10.3389/fimmu.2020.00339 32226426 PMC7080697

[31] Robert C Schachter J Long GV Arance A Ribas A. Pembrolizumab Versus Ipilimumab in Advanced Melanoma. New Engl J Med (2015) 372(26):2521–32. doi: 10.1056/NEJMoa1503093 25891173

[32] Siqi C Jing L Qing L. Bispecific Antibodies in Cancer Immunotherapy. Hum Vaccines Immunotherapeutics (2016) 12(2164):2491–500. doi: 10.1080/21645515.2016.1187802 PMC5084997 27249163

[33] Zhao Qi. Bispecific Antibodies for Autoimmune and Inflammatory Diseases: Clinical Progress to Date. BioDrugs (2020) 34:111–9. doi: 10.1007/s40259-019-00400-2 31916225

[34] Kontermann RE Brinkmann U. Bispecific Antibodies. Drug Discovery Today (2015) 20:838–47. doi: 10.1016/j.drudis.2015.02.008 25728220

[35] Roland EK. Dual Targeting Strategies With Bispecific Antibodies. Mabs (2012) 4(2):182–97. doi: 10.4161/mabs.4.2.19000 PMC3361654 22453100

[36] Labrijn AF Janmaat ML Reichert JM Parren P. Bispecific Antibodies: A Mechanistic Review of the Pipeline. Nat Rev Drug Discov (2019) 18(8):1–24. doi: 10.1038/s41573-019-0028-1 31175342

[37] Francois D Amy C Sharon S Eric T Glenn PK Bing X. HTRF: A Technology Tailored for Drug Discovery - A Review of Theoretical Aspects and Recent Applications. Curr Chem Genomics (2009) 3:22–32. doi: 10.2174/1875397300903010022 20161833 PMC2802762

[38] Khler G Milstein C. Derivation of Specific Antibody-Producing Tissue Culture and Tumor Lines by Cell Fusion. Eur J Immunol (1976) 6:511–9. doi: 10.1002/eji.1830060713 825377

[39] Carter P Ridgway J Zhu Z. Toward the Production of Bispecific Antibody Fragments for Clinical Applications. J Hematother (1995) 4(5):463–70. doi: 10.1089/scd.1.1995.4.463 8581386

[40] Plückthun A Pack P. New Protein Engineering Approaches to Multivalent and Bispecific Antibody Fragments. Immunotechnology (1997) 3(2):83–105. doi: 10.1016/S1380-2933(97)00067-5 9237094

[41] Allen E Jabouille A Rivera LB Lodewijckx I Missiaen R Steri V. Combined Antiangiogenic and Anti-PD-L1 Therapy Stimulates Tumor Immunity Through HEV Formation. Sci Transl Med (2017) 9(385):eaak9679. doi: 10.1126/scitranslmed.aak9679 28404866 PMC5554432

[42] Yi M Jiao D Qin S Chu Q Wu K Li A. Synergistic Effect of Immune Checkpoint Blockade and Anti-Angiogenesis in Cancer Treatment. Mol Cancer (2019) 18(1):60. doi: 10.1186/s12943-019-0974-6 30925919 PMC6441150

[43] Xu YA Dw A Jl A Xy A Hz A. Atezolizumab Plus Bevacizumab for Unresectable Hepatocellular Carcinoma. Lancet Oncol (2020) 21(9):e413. doi: 10.1016/S1470-2045(20)30476-9 32888463

[44] Wallin JJ Bendell JC Funke R Sznol M Korski K Jones S. Atezolizumab in Combination With Bevacizumab Enhances Antigen-Specific T-Cell Migration in Metastatic Renal Cell Carcinoma. Nat Commun (2016) 7:12624. doi: 10.1038/ncomms12624 27571927 PMC5013615

[45] Nomi T Sho M Akahori T Hamada K Kanehiro H Yagita H. Clinical Importance and Therapeutic Potential of Targeting PD-L/PD-1 Pathway in Pancreatic Cancer. Clin Cancer Res (2007) 13(13):2151–7. doi: 10.1158/1078-0432.CCR-06-2746 17404099

[46] Huang M Huang B Li G Zeng S. Apatinib Affect VEGF-Mediated Cell Proliferation, Migration, Invasion *via* Blocking VEGFR2/RAF/MEK/ERK and PI3K/AKT Pathways in Cholangiocarcinoma Cell. BMC Gastroenterol (2018) 18(1):169. doi: 10.1186/s12876-018-0870-3 30400838 PMC6220519

[47] Reinders M Sho M Izawa A Ping W Briscoe DM. Proinflammatory Functions of Vascular Endothelial Growth Factor in Alloimmunity. J Clin Invest (2003) 112(11):1655–65. doi: 10.1172/JCI200317712 PMC281640 14660742

[48] Beckman RA Weiner LM Davis HM. Antibody Constructs in Cancer Therapy: Protein Engineering Strategies to Improve Exposure in Solid Tumors. Cancer (2007) 109(2):170–9. doi: 10.1002/cncr.22402 17154393

[49] Nugent LJ Jain RK. Extravascular Diffusion in Normal and Neoplastic Tissues. Cancer Res (1984) 44(1):238–44. doi: 10.1002/9780470720905.indsub 6197161

[50] Gerlowski LE Jain RK. Microvascular Permeability of Normal and Neoplastic Tissues. Microvasc Res (1986) 31(3):288–305. doi: 10.1016/0026-2862(86)90018-X 2423854

