# Supplementary material for: A Novel Bispecific Antibody Targeting PD-L1 and VEGF With Combined Anti-Tumor Activities
Source: Front Immunol. 2021 Dec 2;12:778978. doi: 10.3389/fimmu.2021.778978 (PMC8678608; doi:10.3389/fimmu.2021.778978)
Supplement: Supplementary file 1 [file DataSheet_1.docx]

**Supplementary Data 1: Methods ane Results of SEC, ICEF and LC-MS**

**1. Liquid Chromatography-Mass Spectrometry (LC-MS) Analysis**

**1.1Method**

Prior to LC-MS analysis, the HB0025 samples were incubated with PNGase-F to remove the N-linked glycans. The HB0025 stock solution was diluted with ultra-pure water to 5 mg/mL. The antibody samples (25 μg each) were injected onto the Agilent Poroshell 300SB-C8 (5-μm particle size) equipped with a 2.1 × 50-mm column (75°C column temperature) and eluted by gradient elution in a reversed-phase chromatography column. The antibodies were eluted from the column with a 15-min gradient (30–40% B, 0.500 mL/min flow rate). The mobile phase was composed of water/acetonitrile/trifluoroacetic acid. The electrospray ionization mass spectrometry (ESI-MS) detection was performed on the samples. The mass-to-charge ratio of the protein sample molecular weight ranged from 2200 to 5000 m/z. MassHunter Qualitative Analysis Software B.07.00 was used to deconvolute the mass spectrum in order to obtain the molecular weight of the protein sample.

**1.2 Results**


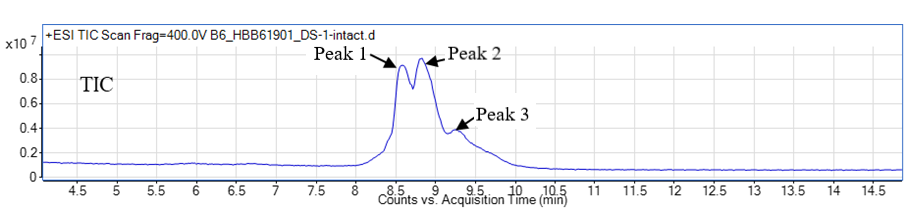


A


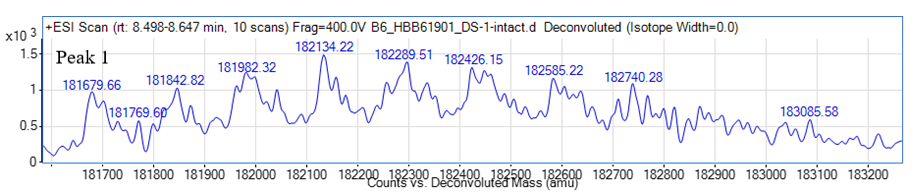


B


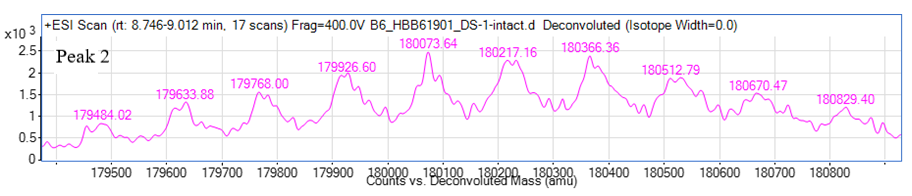


C

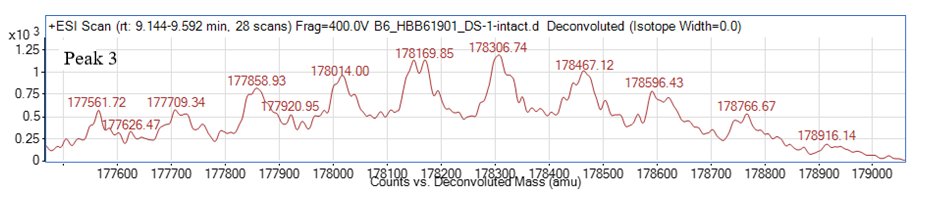


D

**Figure 1. LC-MS analysis of the HB0025 molecular weight**: (A) Intact antibody total ion chromatogram (TIC) in LC-MS analysis. (B)–(D) The corresponding deconvolution spectrum of peak 1, peak 2, and peak 3, respectively, for LC-MS data analyses.

**2. Size Exclusion Chromatography-Ultrahigh Performance Liquid Chromatography (SEC-UPLC) Analysis**

**2.1 Method**

A 50-mM phosphate/300-mM sodium chloride/10% acetonitrile mixture was prepared as the mobile phase. The antibody samples (30 µg) were then injected onto the Waters H-Class ultra-high performance liquid chromatography with TSKgeL UP-SW3000 (4.6 × 300-mm, 2 µm) model column. The flow rate was set to 0.2 mL/min for isocratic elution for 20 min. The main peak and high molecular weight peak contents of raw liquid samples were detected at a wavelength of 280 nm.

**2.2 Results**

After the separation by Size Exclusion Chromatography (SEC), each component was entered into the Multi-angle Light Scatting Detector (MALS). MALS measured the intensity of the scattered light through multiple angles simultaneously. LS, UV, and dRI signals are plotted in red, green, and blue lines, respectively in the below figure.

**
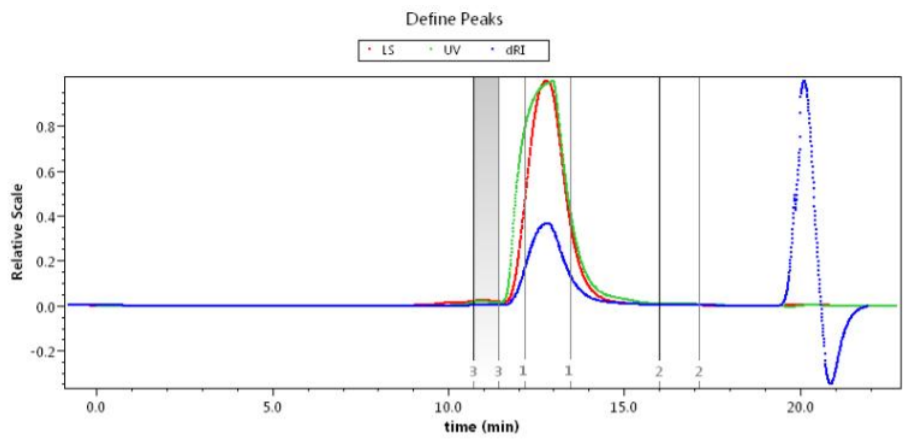
**

**Figure 2. The SEC-MALS chromatography**

Table 1. Calculated molecular weight

| Samples | HMW Peak (Da) | Main Peak (Da) |
| --- | --- | --- |
| 1 | 6.376 × 10^5^ (±0.612%) | 1.804 × 10^5^ (±0.047%) |
| 2 | 6.462 × 10^5^ (±0.618%) | 1.839 × 10^5^ (±0.050%) |

**3. Imaging Capillary Isoelectric Focusing (ICIEF) Analysis**

**3.1 Method**

ICIEF analysis in these studies was performed by using the ProteinSimple/iCE capillary electrophoresis system (ProteinSimple, San Jose, California, USA). The samples were first diluted to 10 mg/mL with deionized water. The sample solution was mixed with Pharmalyte, Methyl Cellulose, and PI Marker. Finally, this mixed solution with 0.2 mg/mL sample concentration was injected. The instrument parameters were as follows: detection wavelength, 280 nm; focusing procedure, 1500V for 1 min, 3000V for 8 min; inject time, 91 s; solution buffer inject time, 136 s.

**3.2 Results**

To identify the charge variant distribution, iCIEF was performed. According to the distribution of isoelectric points, the main spectrum was defined as acid peaks (Figure A, peaks 1–3), main peaks (Figure A, peaks 8–11), and basic peaks (Figure A, peaks 4–7). The overall charge distribution was extremely wide, likely due to the multiple glycosylation sites and the rich sialic acid modification. Further analysis by hydrolyzing HB0025 with α 2-3,6,8,9 neuraminidase A enzyme was performed. The charge heterogeneity distributions of the treated HB0025 sample are shown in Figure B. After hydrolysis, the charge distributions became significantly simpler, and the isoelectric point was increased, confirming that the modification of terminal sialic acid to N-glycoside was the main reason for the complex charge distribution of HB0025. To identify the influence of charge variants on the PD-L1 antibody and VEGF the domain-binding affinity, the three components were collected to conduct the SPR (Biacore) affinity analysis. The results revealed that the acid peak, main peak, and basic peak components had no affinity difference with the control (Table1). Therefore, all the components (i.e., acid peaks, basic peaks, and main peaks) can be considered as the active ingredient of HB0025.


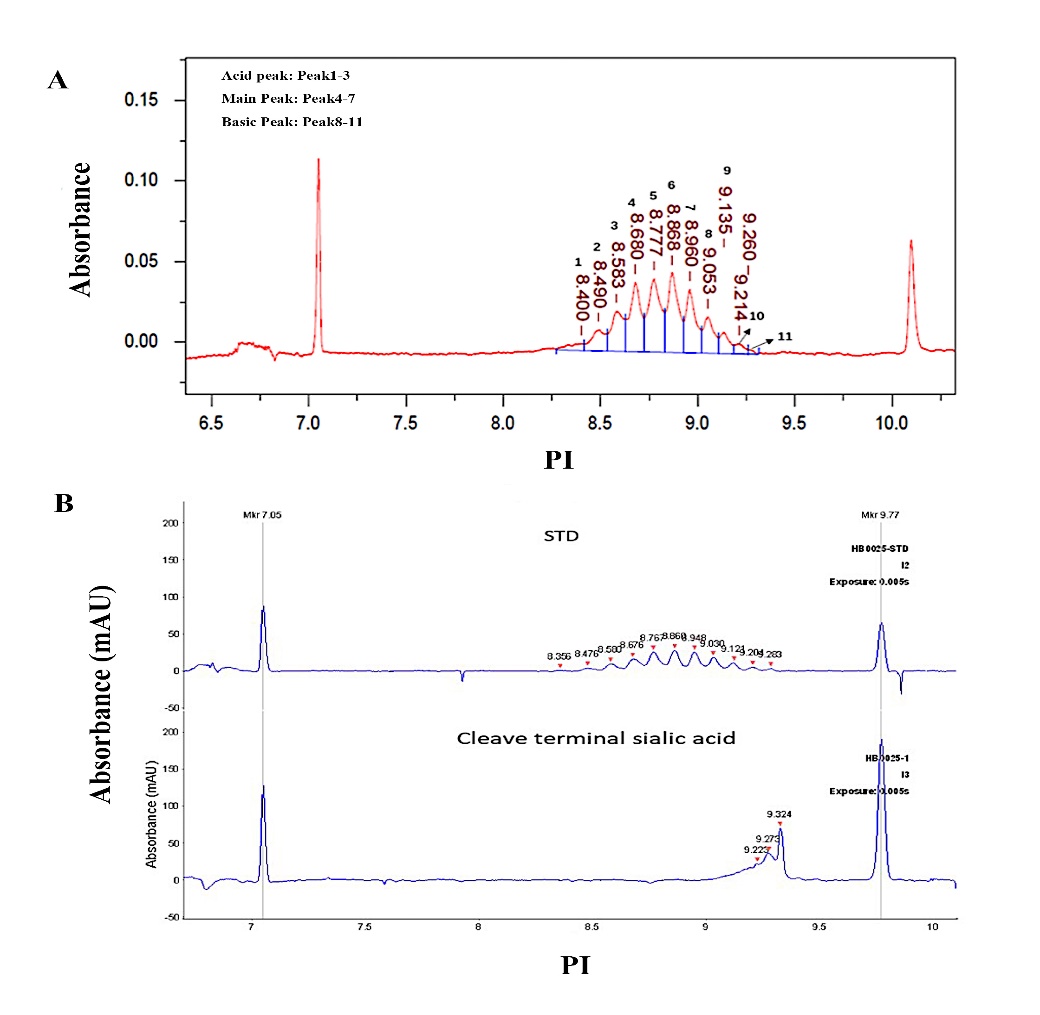


**Figure 3. Analysis of HB0025 charge isomers.** (A) Profiles from cIEF separation of HB0025 stock solution; pI 7.03 and pI 10.33 denote the pI markers. Acid peak: Peaks 1–3, Main Peak: Peaks 4–7, Basic Peak: Peaks 8–11. (B) The charge heterogeneity distribution (iCIEF) of the treated HB0025 sample using α 2-3,6,8,9 neuraminidase A enzyme. pI 7.05 and pI 9.77 denote the pI markers.

Table: Affinity analysis of PD-L1 and VEGF165 by SPR (Biacore) assays.

| Antibody | Component | ka (1/Ms) | kd (1/s) | KD (M) |
| --- | --- | --- | --- | --- |
| PD-L1 | Control | 2.65×10^5^ | 5.77×10^-4^ | 2.18×10^-9^ |
|  | Acid peak | 2.93×10^5^ | 6.55×10^-4^ | 2.24×10^-9^ |
|  | Main peak | 2.86×10^5^ | 6.22×10^-4^ | 2.17×10^-9^ |
|  | Basic peak | 2.83×10^5^ | 6.58×10^-4^ | 2.33×10^-9^ |
| VEGF165 | Control | 2.31×10^7^ | 1.37×10^-4^ | 5.93×10^-12^ |
|  | Acid peak | 1.09×10^7^ | 1.09×10^-4^ | 9.98×10^-12^ |
|  | Main peak | 2.74×10^7^ | 1.57×10^-4^ | 5.72×10^-12^ |
|  | Basic peak | 3.57×10^7^ | 2.25×10^-4^ | 6.29×10^-12^ |

Ka: association constant, Kd: dissociation constant, KD = Kd/Ka.
