# Supplementary figures and images for: A Novel Bispecific Antibody Targeting PD-L1 and VEGF With Combined Anti-Tumor Activities
Source: Front Immunol. 2021 Dec 2;12:778978. doi: 10.3389/fimmu.2021.778978 (PMC8678608; doi:10.3389/fimmu.2021.778978)

Supplementary Figure1: Individual tumor growth curves


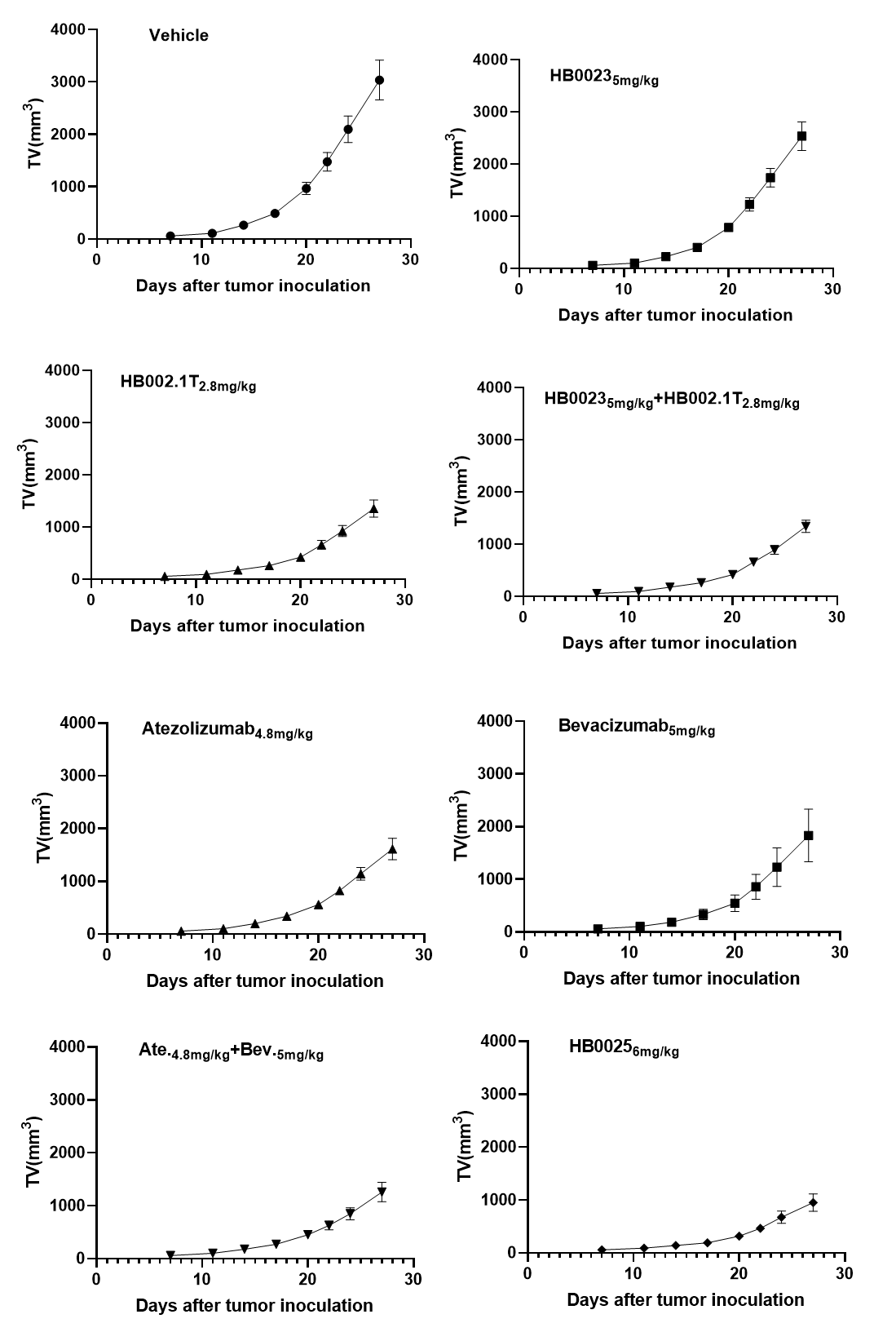

Supplement: Supplementary file 2 [file DataSheet_2.docx]
