# Supplementary material for: A Novel Bispecific Antibody Targeting PD-L1 and VEGF With Combined Anti-Tumor Activities
Source: Front Immunol. 2021 Dec 2;12:778978. doi: 10.3389/fimmu.2021.778978 (PMC8678608; doi:10.3389/fimmu.2021.778978)
Supplement: Supplementary file 3 [file DataSheet_3.docx]

## Supplementary Figure 2. Representative IHC images of tumors

Note: CD3 (yellow), CD4 (purple), CD8 (green), CD31 (red), and DAPI (blue); magnification: 200×

**
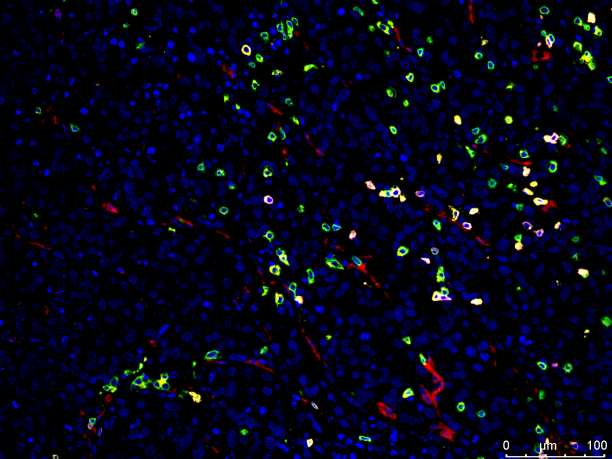

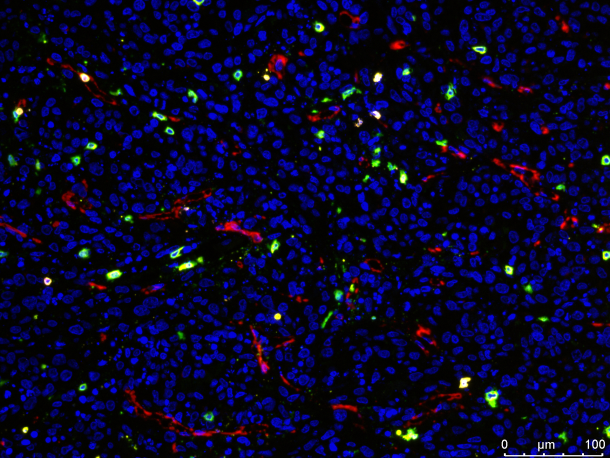
**

**G1-19 G1-24**

**
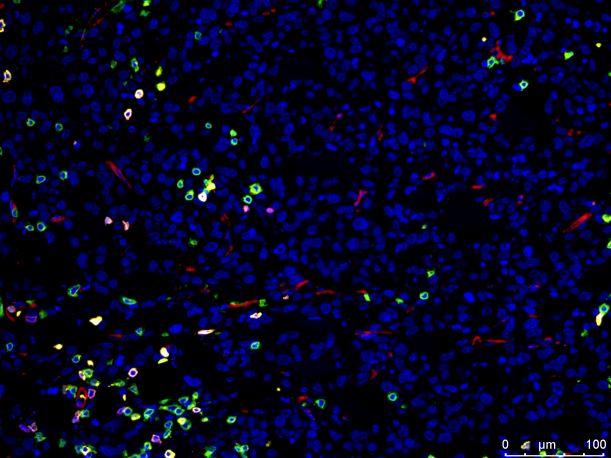

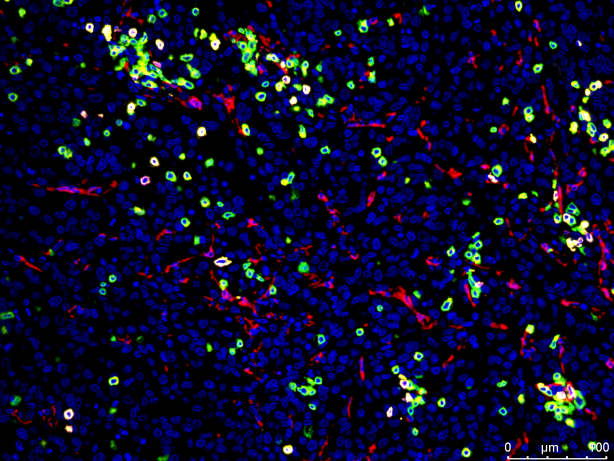
**

**G1-26 G1-37**

**
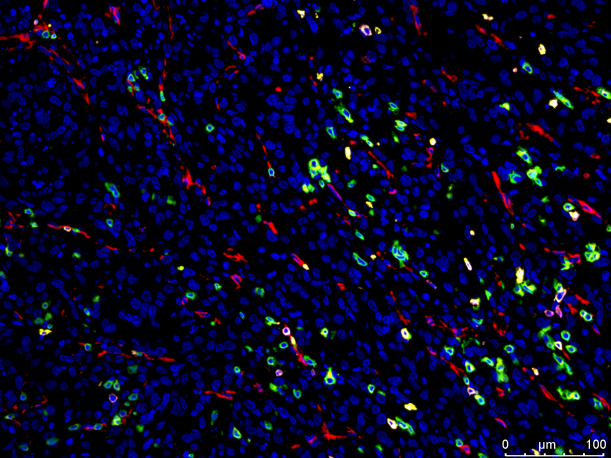

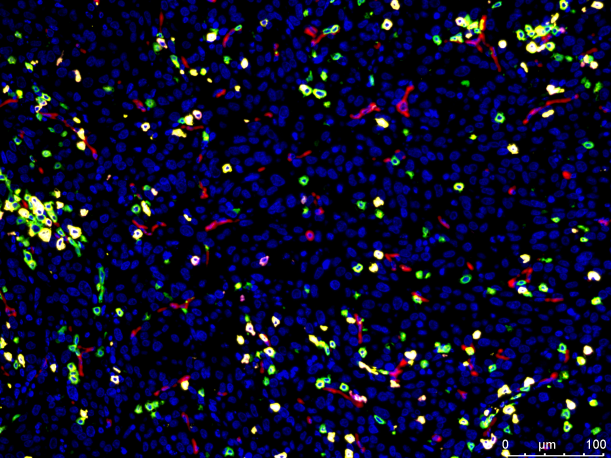
**

**G1-63 G2-18**

**G1:Vehicle**

**
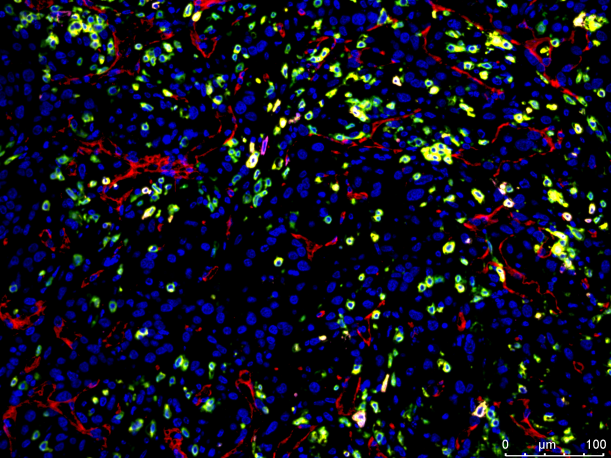

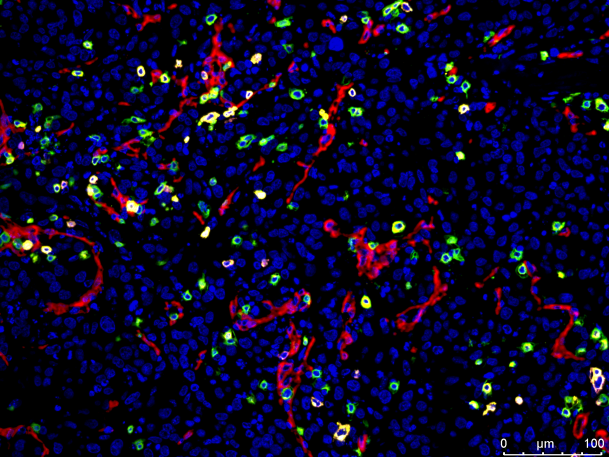
**

**G2-28 G2-32**

**
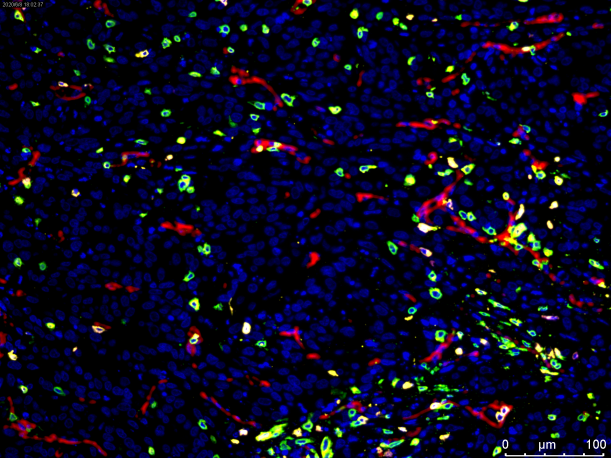

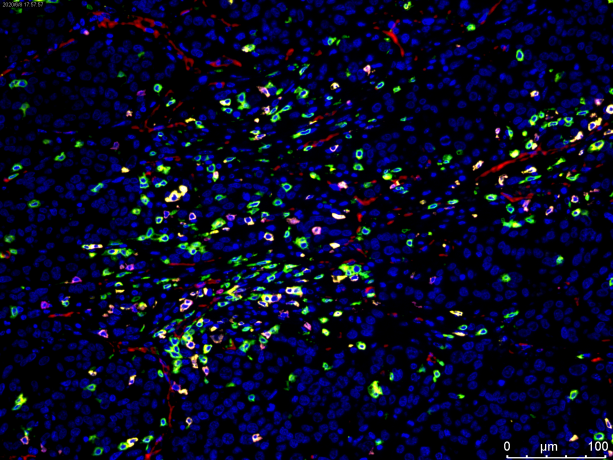
**

**G2-42 G2-46**

**G2: HB0023 5mg/kg**

**
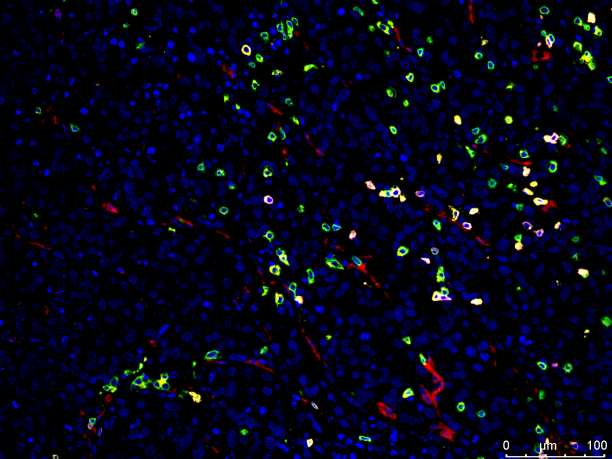

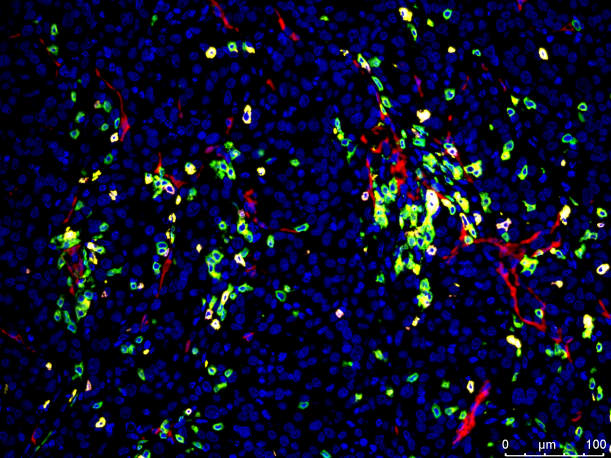
**

**G3-14 G3-15**

**
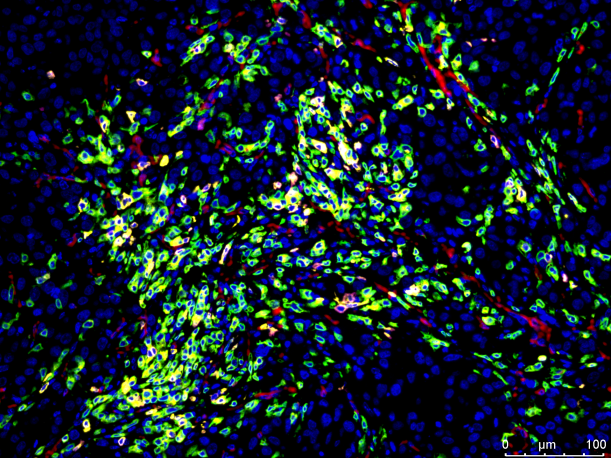

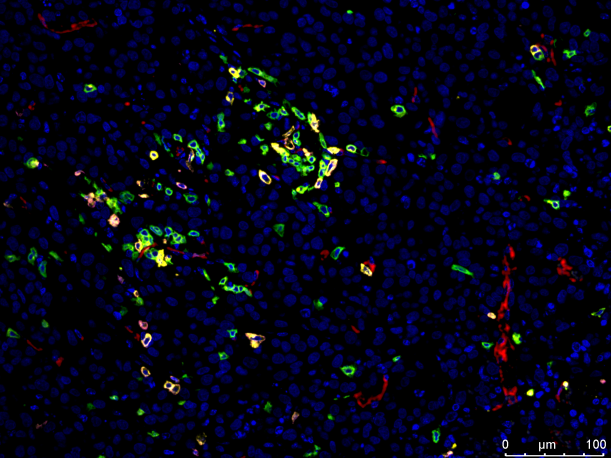
**

**G3-41 G3-45**

**
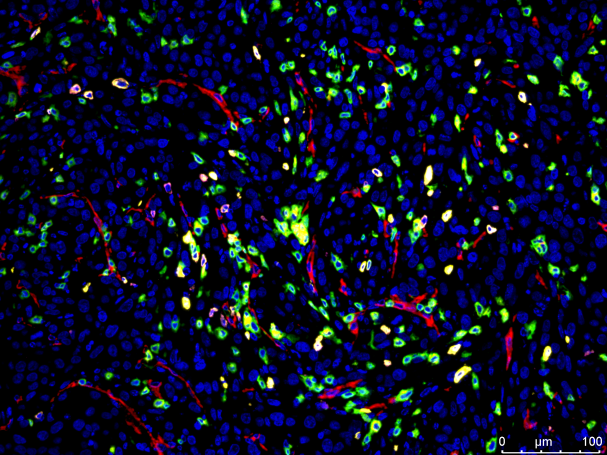
**

**G3-72**

**G3: HB0025 2.8mg/kg**

**
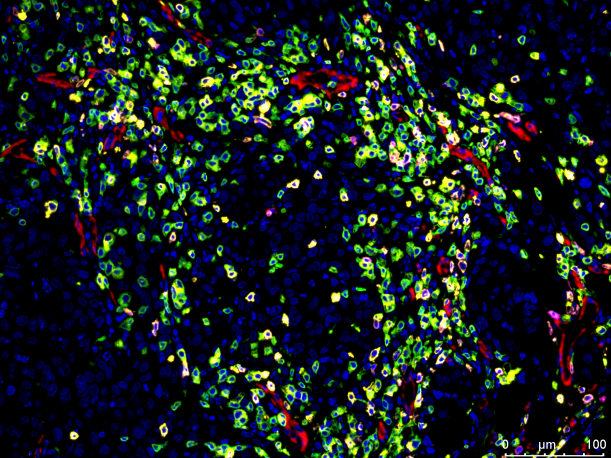
**

**G4-50**

**
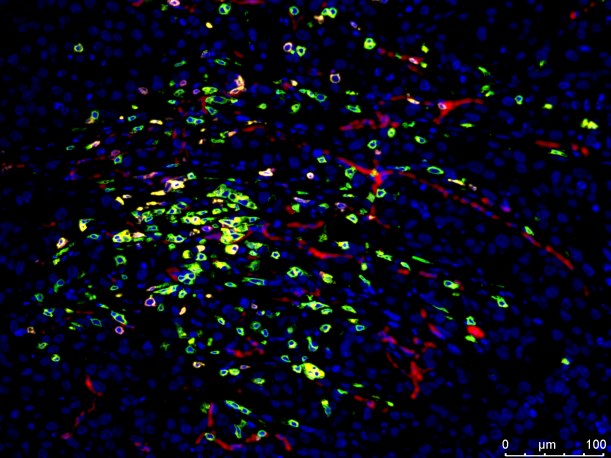

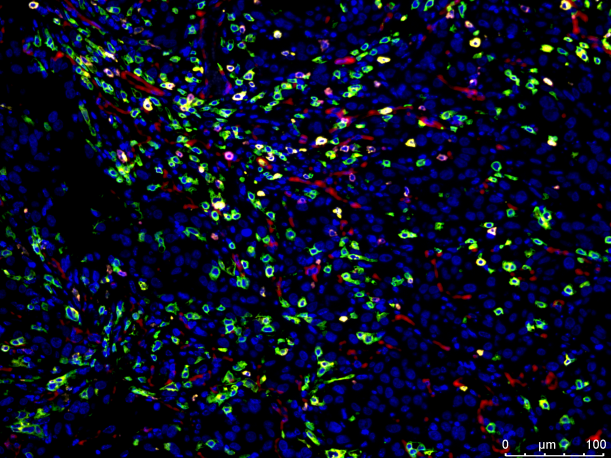
**

**G4-52 G4-59**

**
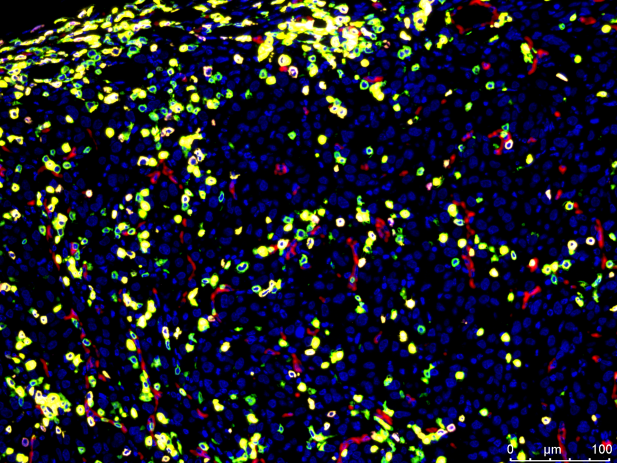

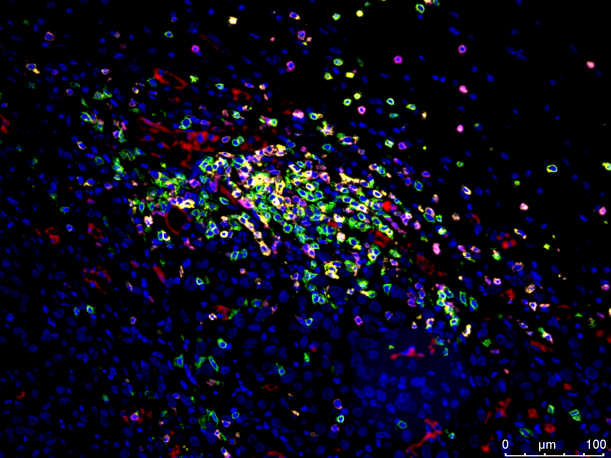
**

**G4-79 G4-89**

**G4: HB0023+HB002.1T 5 mg/kg+2.8 mg/kg**

**
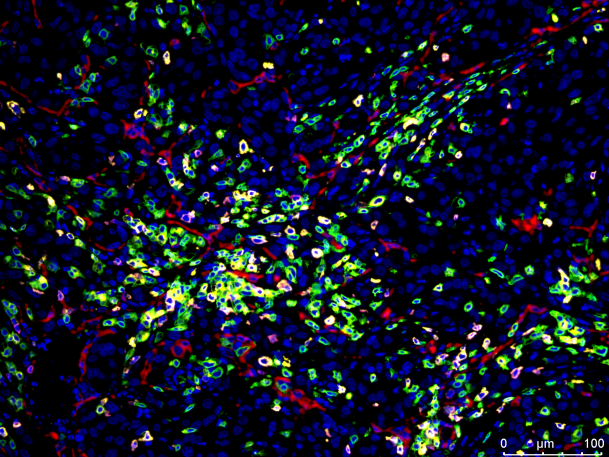

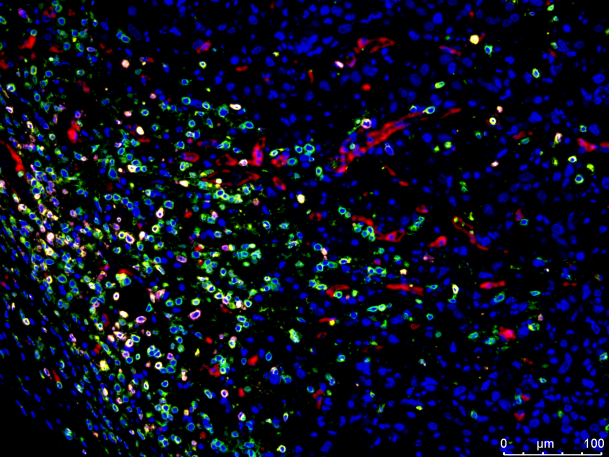
**

**G5-39 G5-43**

**
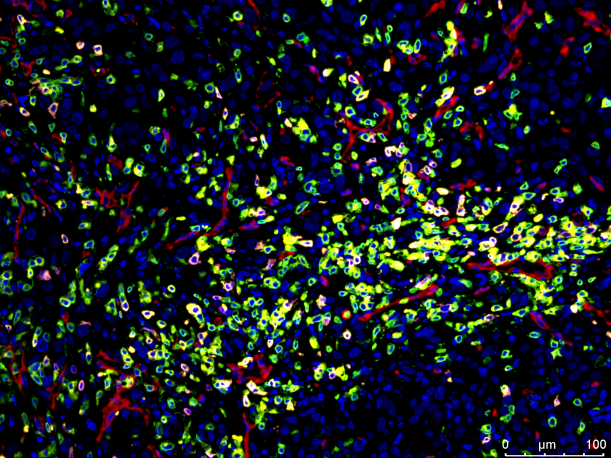

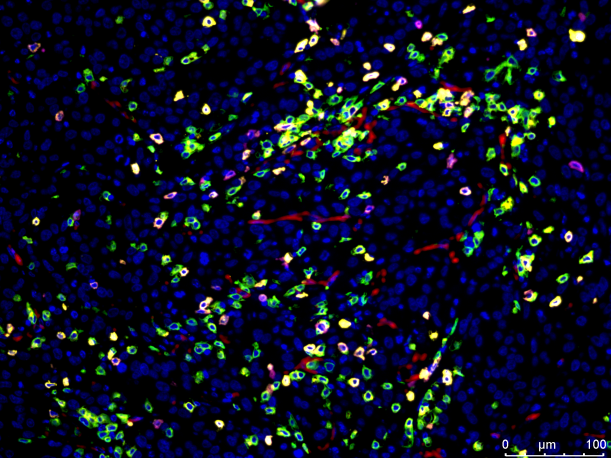
**

**G5-56 G5-58**

**
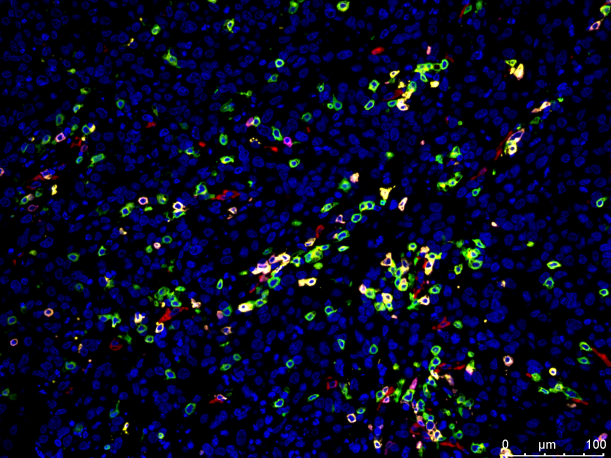
**

**G5-61**

**G5: HB0025 3mg/kg**

**
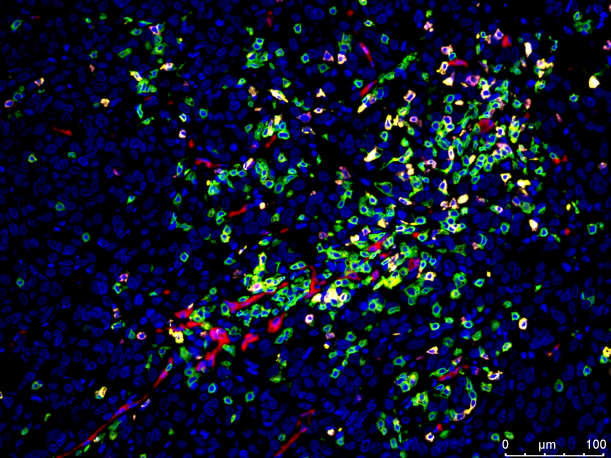
**

**G6-9**

**
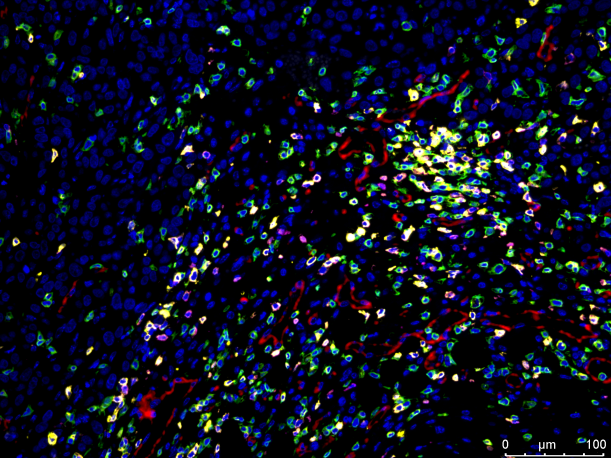

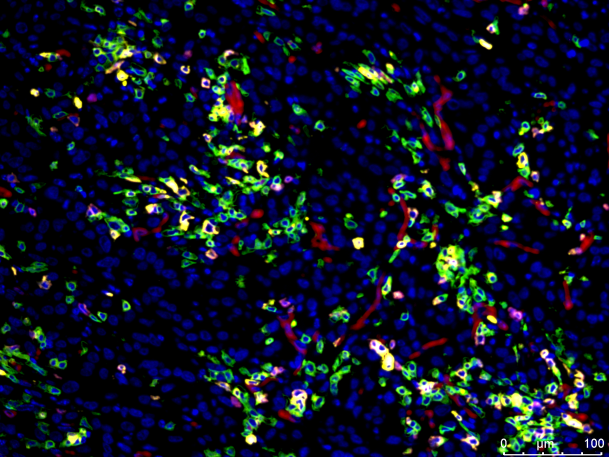
**

**G6-10 G6-20**

**
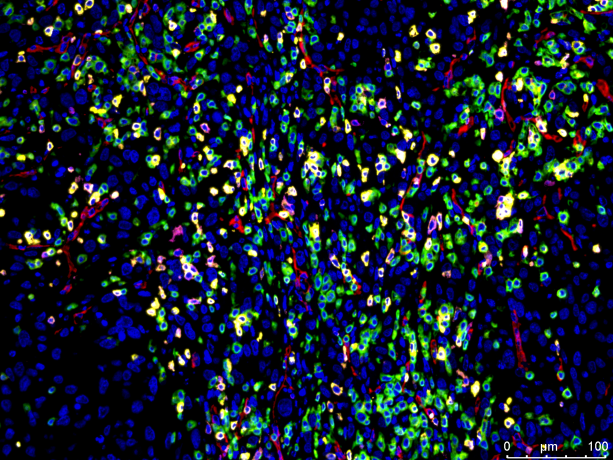

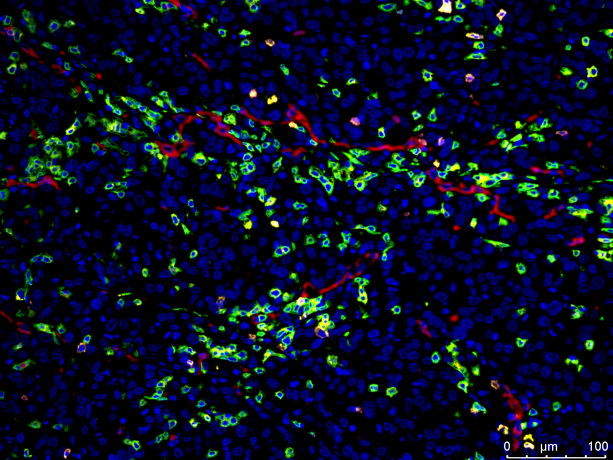
**

**G6-53 G6-76**

**G6: HB0025 6mg/kg**

**
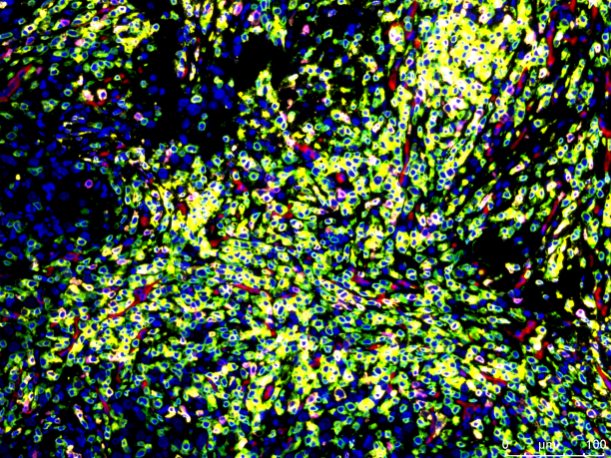

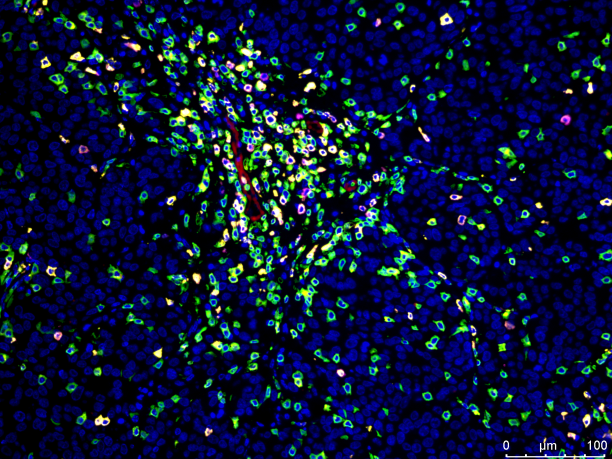
**

**G7-7 G7-33**

**
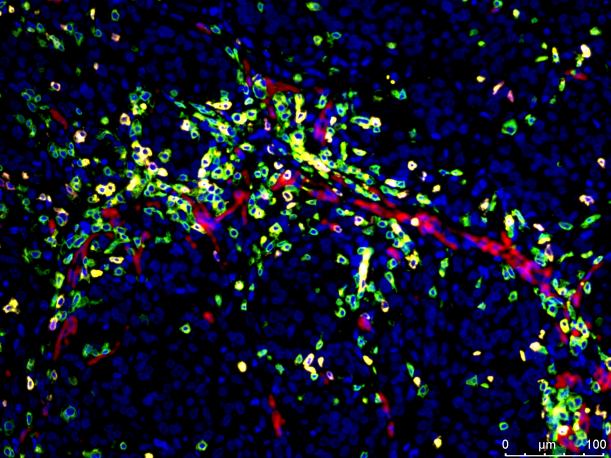

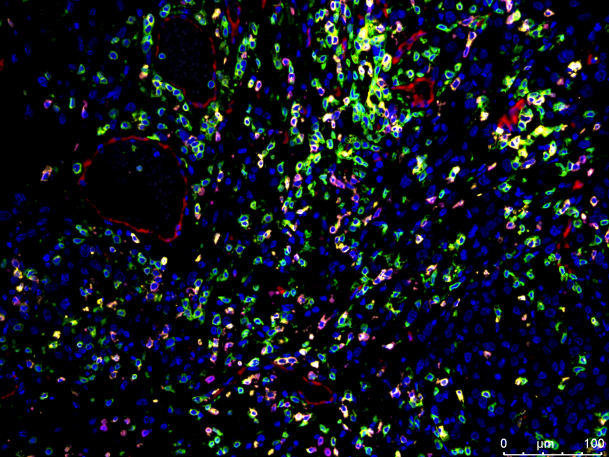
**

**G7-35 G7-51**

**
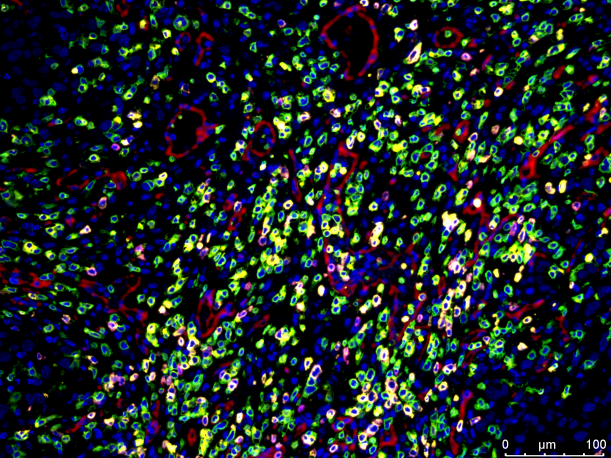
**

**G7-55**

**G7: HB0025 12mg/kg**
