# Supplementary material for: A Novel Bispecific Antibody Targeting PD-L1 and VEGF With Combined Anti-Tumor Activities
Source: Front Immunol. 2021 Dec 2;12:778978. doi: 10.3389/fimmu.2021.778978 (PMC8678608; doi:10.3389/fimmu.2021.778978)
Supplement: Supplementary file 4 [file Table_1.docx]

Supplemental Table 1. Stoichiometric ratio of HB0025 samples binds to PD-L1 and VEGF

| Ligand | Analyte1 | Analyte2 | Analyte1/Ligand Stoichiometric Ratio | Analyte2/Ligand Stoichiometric Ratio |
| --- | --- | --- | --- | --- |
| HB0025 | Human PD-L1 | Human/Cynomolgus VEGF | 1.779 | 1.093 |
|  | Human/Cynomolgus VEGF | Human PD-L1 | 1.242 | 1.735 |
| HB0023 | Human PD-L1 | Human/Cynomolgus VEGF | 1.593 | NB |
|  | Human/Cynomolgus VEGF | Human PD-L1 | NB | 1.610 |
| HB002.1T | Human PD-L1 | Human/Cynomolgus VEGF | NB | 1.138 |
|  | Human/Cynomolgus VEGF | Human PD-L1 | 1.134 | NB |

NB, No binding.
