# Supplementary material for: A Novel Bispecific Antibody Targeting PD-L1 and VEGF With Combined Anti-Tumor Activities
Source: Front Immunol. 2021 Dec 2;12:778978. doi: 10.3389/fimmu.2021.778978 (PMC8678608; doi:10.3389/fimmu.2021.778978)
Supplement: Supplementary file 5 [file Table_2.docx]

## Supplementary Table 2. Raw data of blood flow cytometry (%).

| **Group** | **Animal No.** | **2020/4/24** | **2020/5/14** |
| --- | --- | --- | --- |
|  |  | **hCD45%** | **hCD45%** |
|  | 19 | 1.94% | 43.46% |
| G1 | 24 | 2.40% | 92.72% |
| Vehicle | 26 | 1.66% | 67.94% |
| i.v. | 37 | 3.25% | 50.91% |
| tiw x 8 doses | 63 | 2.74% | 82.39% |
|  | 70 | 1.41% | 63.84% |
|  | 99 | 3.87% | 59.75% |
|  | 139 | 1.62% | 66.21% |
|  | 146 | 1.61% | 67.41% |
|  | 154 | 6.32% | 45.90% |
|  | AV | 2.68% | 64.05% |
|  | SE | 0.48% | 4.87% |
|  | 18 | 3.87% | 55.37% |
| G2 | 28 | 2.31% | 26.38% |
| HB0023 | 32 | 1.84% | 52.58% |
| 5 mg/kg | 42 | 6.07% | 62.03% |
| i.v. | 46 | 4.81% | 47.86% |
| tiw x 8 doses | 54 | 1.73% | 41.56% |
|  | 83 | 2.76% | 28.40% |
|  | 88 | 1.97% | 66.38% |
|  | 90 | 3.56% | 74.25% |
|  | 94 | 2.68% | 51.25% |
|  | AV | 3.16% | 50.61% |
|  | SE | 0.45% | 4.87% |
|  | 14 | 3.17% | 71.19% |
| G3 | 15 | 4.02% | 53.10% |
| HB002.1T | 41 | 4.43% | 62.04% |
| 2.8 mg/kg | 45 | 2.92% | 47.09% |
| i.v. | 72 | 2.56% | 67.92% |
| tiw x 8 doses | 100 | 3.22% | 64.00% |
|  | 101 | 1.60% | 76.40% |
|  | 110 | 1.77% | 60.65% |
|  | 125 | 1.91% | 67.15% |
|  | 142 | 2.58% | 66.81% |
|  | AV | 2.82% | 63.64% |
|  | SE | 0.30% | 2.70% |
|  | 50 | 3.92% | 46.58% |
| G4 | 52 | 2.57% | 31.92% |
| HB0023+HB002.1T | 59 | 6.38% | 64.90% |
| 5 mg/kg+2.8 mg/kg | 79 | 3.11% | 44.98% |
| i.v.+i.v. | 89 | 2.74% | 67.58% |
| tiw x 8 doses | 96 | 2.66% | 74.22% |
|  | 106 | 2.73% | 64.21% |
|  | 144 | 4.88% | 74.71% |
|  | 127 | 1.65% | 56.77% |
|  | 129 | 4.68% | 37.42% |
|  | AV | 3.53% | 56.33% |
|  | SE | 0.45% | 4.83% |
|  | 39 | 2.48% | 60.37% |
| G5 | 43 | 2.08% | 38.06% |
| HB0025 | 56 | 1.95% | 40.97% |
| 3 mg/kg | 58 | 2.73% | 61.79% |
| i.v. | 61 | 2.08% | 40.35% |
| tiw x 8 doses | 62 | 2.09% | 67.44% |
|  | 138 | 3.21% | 72.76% |
|  | 74 | 4.21% | 77.04% |
|  | 104 | 4.59% | 26.84% |
|  | 131 | 1.99% | 70.70% |
|  | AV | 2.74% | 55.63% |
|  | SE | 0.30% | 5.54% |
|  | 9 | 1.32% | 33.03% |
| G6 | 10 | 3.00% | 57.19% |
| HB0025 | 20 | 2.25% | 33.29% |
| 6 mg/kg | 159 | 2.44% | 65.40% |
| i.v. | 53 | 3.46% | 37.14% |
| tiw x 8 doses | 76 | 1.44% | 57.57% |
|  | 78 | 5.00% | 38.71% |
|  | 82 | 1.64% | 94.19% |
|  | 86 | 3.08% | 43.27% |
|  | 114 | 1.46% | 78.46% |
|  | AV | 2.51% | 53.83% |
|  | SE | 0.37% | 6.56% |
|  | 7 | 2.90% | 80.31% |
| G7 | 33 | 5.57% | 76.38% |
| HB0025 | 35 | 3.01% | 65.94% |
| 12 mg/kg | 51 | 4.40% | 60.62% |
| i.v. | 55 | 2.29% | 33.24% |
| tiw x 8 doses | 65 | 5.09% | 35.31% |
|  | 81 | 2.20% | 50.92% |
|  | 85 | 5.02% | 64.94% |
|  | 91 | 1.92% | 22.08% |
|  | 134 | 2.09% | 59.39% |
|  | AV | 3.45% | 54.91% |
|  | SE | 0.45% | 6.08% |
|  | 1 | 1.59% | 59.54% |
| G8 | 16 | 2.05% | 74.71% |
| Bevacizumab | 38 | 2.23% | 59.18% |
| 5 mg/kg | 64 | 2.46% | 71.14% |
| i.v. | 66 | 1.44% | 64.06% |
| tiw x 8 doses | 67 | 3.62% | 99.27% |
|  | 80 | 1.72% | 62.45% |
|  | 149 | 2.30% | 64.80% |
|  | 98 | 2.10% | 69.12% |
|  | 113 | 3.71% | 44.08% |
|  | AV | 2.32% | 66.84% |
|  | SE | 0.25% | 4.47% |
|  | 5 | 1.42% | 61.40% |
| G9 | 29 | 1.39% | 55.92% |
| Atezolizumab | 48 | 2.30% | 51.94% |
| 4.8 mg/kg | 73 | 3.87% | 50.32% |
| i.v. | 77 | 2.84% | 50.71% |
| tiw x 8 doses | 92 | 1.59% | 70.29% |
|  | 105 | 3.93% | 22.98% |
|  | 123 | 2.24% | 61.03% |
|  | 130 | 2.68% | 50.06% |
|  | 143 | 3.86% | 57.14% |
|  | AV | 2.61% | 53.18% |
|  | SE | 0.32% | 3.92% |
|  | 12 | 2.30% | 47.50% |
| G10 | 22 | 2.56% | 27.95% |
| Atezolizumab + Bevacizumab | 27 | 1.82% | 51.26% |
| 5 mg/kg+4.8 mg/kg | 47 | 1.64% | 33.96% |
| i.v.+i.v. | 57 | 1.41% | 30.38% |
| tiw x 8 doses | 75 | 1.44% | 34.72% |
|  | 93 | 5.60% | 60.56% |
|  | 102 | 1.55% | 72.63% |
|  | 111 | 2.81% | 55.46% |
|  | 116 | 4.82% | 28.91% |
|  | AV | 2.60% | 44.33% |
|  | SE | 0.46% | 4.88% |
