# Supplementary material for: A Novel Bispecific Antibody Targeting PD-L1 and VEGF With Combined Anti-Tumor Activities
Source: Front Immunol. 2021 Dec 2;12:778978. doi: 10.3389/fimmu.2021.778978 (PMC8678608; doi:10.3389/fimmu.2021.778978)
Supplement: Supplementary file 6 [file Table_3.docx]

Supplementary Table 3. Tumor Volume Comparison between Different Groups

| **Group** | **Treatment** | **n** | **TV(mm^3^)^a^** | | **TGI_TV_(%)** | ***P ^b^*** | ***P ^c^*** | ***P ^d^*** | ***P ^e^*** |
| --- | --- | --- | --- | --- | --- | --- | --- | --- | --- |
|  |  |  | **at randomization** | **at termination** |  |  |  |  |  |
| 1 | Vehicle | 10 | 59±2 | 3034±380 | - | - | - | - | - |
| 2 | HB0023 | 10 | 60±2 | 2536±274 | 17% | 0.429 | 0.007 | 0.000 | - |
| 3 | HB002.1T | 10 | 60±3 | 1353±165 | 54% | 0.000 | 0.945 | 0.015 | - |
| 4 | HB0023 | 10 | 59±2 | 1340±119 | 55% | 0.001 | - | - | 0.012 |
|  | HB002.1T |  |  |  |  |  |  |  |  |
| 5 | HB0025 3mg/kg | 10 | 58±2 | 964±101 | 66% | 0.000 | 0.229 | - | - |
| 6 | HB0025 6mg/kg | 10 | 59±2 | 952±163 | 69% | 0.000 | - | - | - |
| 7 | HB0025 12mg/kg | 10 | 59±2 | 955±128 | 67% | 0.000 | 0.311 | - | - |
| 8 | Bevacizumab | 10 | 59±2 | 1831±158 | 38% | 0.028 | 0.073 | 0.000 | - |
| 9 | Atezolizumab | 10 | 59±2 | 1614±203 | 46% | 0.005 | 0.240 | 0.002 | - |
| 10 | Bevacizumab | 10 | 59±2 | 1257±186 | 59% | 0.000 | - | - | 0.042 |
|  | Atezolizumab |  |  |  |  |  |  |  |  |

Note: ^a^. data expressed as mean±SEM; ^b.^ compared Vehicle group with all treatment groups; ^c.^ compared G4 with G2 and G3, compared G6 with G5 and G7, compared G10 with G8 and G9; ^d.^ compared G6 with G2, G3, G8 and G9; ^e.^ compared G6 with G4 and G10.
