# Supplementary material for: A Novel Bispecific Antibody Targeting PD-L1 and VEGF With Combined Anti-Tumor Activities
Source: Front Immunol. 2021 Dec 2;12:778978. doi: 10.3389/fimmu.2021.778978 (PMC8678608; doi:10.3389/fimmu.2021.778978)
Supplement: Supplementary file 7 [file Table_4.docx]

## Supplementary Table 4. Raw data of tumor IHC scoring

| **Group** | **Animal No.** | **CD3+ Cell (%)** | **CD4+ Cell (%)** | **CD8+ Cell (%)** | **CD31+ Vessel** |
| --- | --- | --- | --- | --- | --- |
| G1 | 19 | 16.4 | 7.2 | 14.9 | 28.0 |
| Vehicle | 24 | 5.5 | 2.1 | 4.9 | 45.0 |
| i.v. | 26 | 10.4 | 3.6 | 8.9 | 39.0 |
| tiw x 8 doses | 37 | 11.5 | 3.9 | 10.0 | 76.0 |
|  | 63 | 11.2 | 3.7 | 11.1 | 64.0 |
|  | AV | 11.0 | 4.1 | 10.0 | 50.4 |
|  | SE | 1.7 | 0.8 | 1.6 | 8.7 |
| G2 | 18 | 20.1 | 9.0 | 12.3 | 49.0 |
| HB0023 | 28 | 28.6 | 4.8 | 25.1 | 65.0 |
| 5 mg/kg | 32 | 11.8 | 4.2 | 10.2 | 51.0 |
| i.v. | 42 | 21.5 | 6.9 | 18.7 | 41.0 |
| tiw x 8 doses | 46 | 23.3 | 9.8 | 17.4 | 39.0 |
|  | AV | 21.1 | 6.9 | 16.7 | 49.0 |
|  | SE | 2.7 | 1.1 | 2.6 | 4.6 |
| G3 | 14 | 27.3 | 8.0 | 26.1 | 37.0 |
| HB002.1T | 15 | 21.0 | 6.6 | 18.3 | 36.0 |
| 2.8 mg/kg | 41 | 43.7 | 8.6 | 42.6 | 49.0 |
| i.v. | 45 | 21.9 | 10.4 | 19.1 | 26.0 |
| tiw x 8 doses | 72 | 19.0 | 5.4 | 18.9 | 47.0 |
|  | AV | 26.6 | 7.8 | 25.0 | 39.0 |
|  | SE | 4.5 | 0.9 | 4.6 | 4.2 |
| G4 | 50 | 47.5 | 13.7 | 39.6 | 32.0 |
| HB0023+HB002.1T | 52 | 30.4 | 10.9 | 25.1 | 34.0 |
| 5 mg/kg+2.8 mg/kg | 59 | 29.0 | 7.8 | 28.2 | 51.0 |
| i.v.+i.v. | 79 | 42.8 | 12.5 | 29.7 | 55.0 |
| tiw x 8 doses | 89 | 39.3 | 23.4 | 23.1 | 35.0 |
|  | AV | 37.8 | 13.7 | 29.1 | 41.4 |
|  | SE | 3.6 | 2.6 | 2.9 | 4.8 |
| G5 | 39 | 36.6 | 13.5 | 33.4 | 54.0 |
| HB0025 | 43 | 35.7 | 10.1 | 32.9 | 42.0 |
| 3 mg/kg | 56 | 44.0 | 12.7 | 35.9 | 60.0 |
| i.v. | 58 | 34.3 | 13.9 | 27.9 | 25.0 |
| tiw x 8 doses | 61 | 17.9 | 8.0 | 13.5 | 23.0 |
|  | AV | 33.7 | 11.6 | 28.7 | 40.8 |
|  | SE | 4.3 | 1.1 | 4.0 | 7.5 |
| G6 | 9 | 30.5 | 11.3 | 30.0 | 27.0 |
| HB0025 | 10 | 40.9 | 17.7 | 33.1 | 33.0 |
| 6 mg/kg | 20 | 37.5 | 15.8 | 34.7 | 31.0 |
| i.v. | 53 | 41.9 | 14.5 | 35.8 | 55.0 |
| tiw x 8 doses | 76 | 27.3 | 3.7 | 23.1 | 24.0 |
|  | AV | 35.6 | 12.6 | 31.3 | 34.0 |
|  | SE | 2.9 | 2.5 | 2.3 | 5.5 |
| G7 | 7 | 82.0 | 19.0 | 69.6 | 55.0 |
| HB0025 | 33 | 32.3 | 10.0 | 30.1 | 3.0 |
| 12 mg/kg | 35 | 41.7 | 11.6 | 36.0 | 36.0 |
| i.v. | 51 | 49.7 | 21.1 | 38.1 | 21.0 |
| tiw x 8 doses | 55 | 54.4 | 15.3 | 46.3 | 54.0 |
|  | AV | 52.0 | 15.4 | 44.0 | 33.8 |
|  | SE | 8.4 | 2.1 | 6.9 | 9.9 |

Note: The percentage was the numbers of CD3^+^/CD4^+^/CD8^+^ cells divided by the total number of DAPI dyed cells.
